# Palliative care guidelines for the management of HIV-infected people in South Africa

**DOI:** 10.4102/sajhivmed.v20i1.1013

**Published:** 2019-12-13

**Authors:** David C. Spencer, René Krause, Theresa Rossouw, Mahomed-Yunus S. Moosa, Selma Browde, Esnath Maramba, Lauren Jankelowitz, Muhangwi B. Mulaudzi, Mpho Ratishikana-Moloko, Oluwarotimi F. Modupe, Adam Mahomed

**Affiliations:** 1 Division of Infectious Diseases, Department of Medicine, Helen Joseph Hospital, University of the Witwatersrand, Johannesburg, South Africa; 2 Department of Family Medicine, University of Cape Town, Cape Town, South Africa; 3 Department of Immunology, University of Pretoria, Pretoria, South Africa; 4 Department of Infectious Diseases, Nelson Mandela School of Medicine, University of KwaZulu-Natal, Durban, South Africa; 5 Community Action NGO/NPO, Johannesburg, South Africa; 6 Clinical Unit, Council for Medical Schemes, Pretoria, South Africa; 7 Southern African HIV Clinicians Society, Johannesburg, South Africa; 8 Phomolong Medical Centre, Rustenburg, South Africa; 9 Chris Hani Baragwanath Academic Hospital, University of the Witwatersrand, Johannesburg, South Africa; 10 Council for Medical Schemes, Pretoria, South Africa; 11 Department of Internal Medicine, Charlotte Maxake Johannesburg Academic Hospital, Johannesburg, South Africa

## Overview and summary

### Introduction: What is palliative care and what are its essential elements?

#### Definition: Palliative care is:

- *Care* that places the relief of suffering at its core, affirms life and does not hasten nor postpone death, but regards dying as a normal process, according to World Health Organization (WHO).
- *Care* that is individualised: the person or patient is its core focus. The recipient is someone who has been diagnosed with a chronic, life-threatening illness that is no longer responsive to curative treatment, for example, cancer, irreversible end-stage end-organ failure, HIV infection and related disorders unresponsive to available treatment, et cetera.
  ■ ‘End-of-life’ care is an aspect of palliative care that specifically refers to the care of persons estimated to have a life expectancy of ≤ 12 months, according to the National Council for Palliative Care, United Kingdom.
- *Care* that is directed towards the control of distressing symptoms including the relief of pain:
  ■ ‘Total Pain’ – this concept refers to pain that cannot be adequately controlled without addressing its contributory factors, namely, physical, emotional, social and spiritual factors.
  ■ The opioid-use crisis – inappropriate opioid use is a major contributor to the opioid addiction crisis currently reported from high- and middle-income countries. *Under-use of and insufficient access to opioids* however characterises opioid use in Africa and other low-income countries. Palliative care offers appropriate access to opioids without the risk of addiction and within the context of a professionally competent team. (Knaul et al.).^42^
- *Care* that is provided by a team. The team is multidisciplinary and comprises nurses, doctors, paramedical persons, for example, physiotherapists, counsellors and accredited members of the religious community. The patient and their personal support network (e.g. family, partner and friends) are advisors to the team and receive support from the team. The team has a leader who takes responsibility for the totality of care, plans specific therapy, prescribes medication, completes medico-legal forms, et cetera. This is usually a medical doctor.
  ■ *Team care* is intended to integrate the medical, practical, psychological and spiritual aspects of care in a system that promotes as active a lifestyle as possible until death. Team care provides support for the patient’s family or partner, et cetera, during the illness and through the time of bereavement.
- *Care* that is not restricted by the patient’s age and is not restricted to a particular access point, such as a local clinic, district hospital or tertiary level medical centre. The 2017 National Draft Policy Framework and Strategy Paper on Palliative Care, Department of Health, South Africa (SA), envisages access to palliative care for all South Africans who are in need. (Comment: These remarks from the National Framework Paper are aspirational. Few public sector facilities offer access to palliative care at this time.)

## Do HIV-infected South Africans need palliative care? Figure 1

Human immunodeficiency virus infection is incurable. About 770 000 people died of HIV worldwide in 2018. More than two-thirds of these died in Africa (UNAIDS Global Aids Update 2019). Although Statistics South Africa has recorded some improvement in the overall survival, HIV-related levels of morbidity and mortality remain high. Mortality is greatest among those not on antiretroviral therapy (ART), that is, either naïve to ART or those who have stopped taking medication and are outside of care. Mortality is also high in the first year after the start of ART. Of South Africa’s 7.97 million people living with HIV (PLWHIV) in 2019, only 4.94 million are on ART. A *detectable viral load while on ART* is usually a sign of treatment failure or poor viral control. These persons are also at increased risk of HIV-related morbidity and mortality.

**FIGURE 1:**
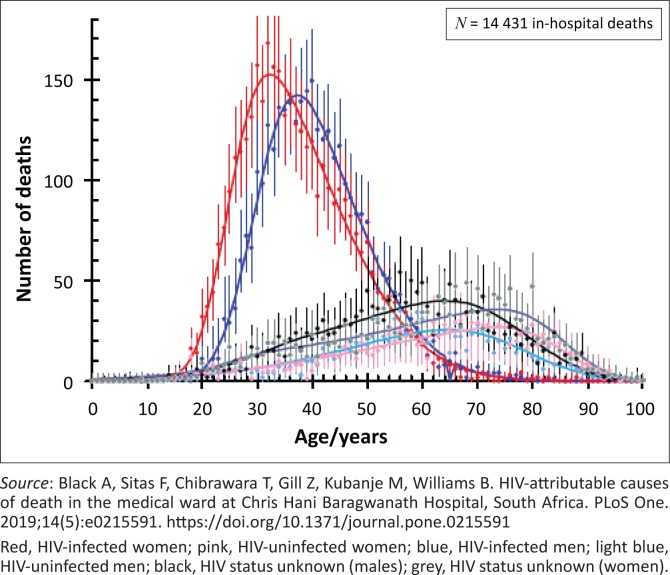
The number of deaths by age and HIV-status of men and women admitted to the Chris Hani Baragwanath Hospital, Soweto, 2006–2009.^8^

## Models of palliative care

- *No palliative care*: Access to formal palliative care within either the public or private health sector in much of Africa including SA, is extremely limited. Few currently access this care (see Figure 3 and Appendix 1).
- *The traditional model of palliative care*: In this scenario, curative care and palliative care are available to the patient at the time of diagnosis. Curative care is initially given priority. But when curative options are exhausted, palliation offers an alternative approach, one that grows in importance as time passes. Despite four decades of HIV research, a cure remains out of reach. In these circumstances, health facilities and healthcare workers must be trained to provide palliative care to those in need of it.
- *The long-term model of HIV palliative care*: In this scenario, ART, prophylactic trimethoprim-sulfamethoxazole, isoniazid (INH) and vaccination against influenza, the pneumococcus, hepatitis B and regular clinic visits form the routine background of care. From time to time, this routine is interrupted by disease. This necessitates a shift in the emphasis of care. Palliation becomes an option for those with incurable conditions, such as a central nervous system (CNS) lymphoma, disabling stroke, renal and liver failure, et cetera. For PLWHIV who live longer, the diseases of old age, for example, chronic lung conditions, diabetes, cardiovascular disease, bone fragility, neurocognitive decline, and the non-AIDS defining cancers, emerge. Indeed, these conditions generally appear a decade or more before identical disease affects uninfected peers (see Figures 4, 5 and 6).

## Formal assessment of the need for palliative care

Limited resources in the face of huge demand necessitate an equitable system of patient triage. With regard to the private funding of costly care options, such as hospice admission and home nursing, these guidelines recommend the SPICT^Tm^ Tool (Appendix 2) and the VACS SCORE criteria (Appendix 3) to be followed as a guide to eligibility. These assessment tools are accredited internationally and with minor adaptation can be used in the South African context.

## The symptomatic management of HIV-infected people receiving palliative care

The core activity of the palliative care team and its clinician(s) is the relief of suffering. Pain, particularly chronic pain lasting ≥ 3 months, is experienced by the majority of PLWHIV before death. A formal approach to the assessment of both acute and chronic pain underlies its successful management (see Table 2). Pain control is not always achieved. However, it is more likely if theoretic knowledge is supplemented with bedside experience. In this regard, the *2017 Guideline on the Management of Chronic Pain* from the HIV Division of the Infectious Diseases Society of America (IDSA), discusses clinical ‘evidence-based’ support for approaches to HIV-related pain syndromes: this is summarised in the ‘Managing the HIV sick’ section. The analgesic drugs are presented in Table 3. Table 3-A6 (Appendix 4) outlines common drug–drug interactions between the antiretrovirals and frequently used analgesics. Additional symptoms such as breathlessness (dyspnoea) and fatigue (weakness) are mentioned in the remainder of ‘Managing the HIV sick’ section. When the natural course of a disease cannot be reversed, kindness, a safe place, food, a clean bed and good symptom control provide the best environment possible for the end of life.

## Cancer care in the HIV-infected patient: Palliative care

The ‘hidden cancer epidemic’ refers to the growing number of HIV-infected cancer patients in Africa. Cancer and the HIV epidemic are interlinked. Uncontrolled plasma (HIV) viral load increases the risk of malignancy. AIDS-defining malignancies were described in the 1980s and 1990s but are still widespread throughout sub-Saharan Africa today. In the era of ART, the risk of non-AIDS defining cancers is also increasing. Universal Test and Treat (UTT) and the 90-90-90 by 2020 initiatives of the WHO and the UNAIDS, will help to reduce risk in the long term if successful.

**TABLE 1:** Causes of death at post-mortem examination of 39 HIV-infected patients of the Charlotte Maxeke Johannesburg Academic Hospital, January–December 2009.^9^

Causes of death by category and/or organism†	All deaths (*N* = 39)		Pre-ART deaths (*N* = 14)		Early-ART deaths (*N* = 15)		Late-ART deaths (*N* = 10)	
	*n*	%	*n*	%	*n*	%	*n*	%
Mycobacterial	27.0	69	8	57	13	87	6	60
Bacterial	13.0	33	5	36	6	40	2	20
Fungal	8.2	21	3	21	4	27	1	10
Viral, not HIV	3.0	8	2	14	0	0	1	10
Neoplasm	10.0	26	3	21	3	20	4	40
Organ dysfunction‡	10.0	26	1	7	5	33	4	40
IRIS	11.0	28	-	-	11	73	-	-
Diagnosis unsuspected at death§	19.0	49	7	50	8	53	4	40
Unexplained	5.0	13	4	29	1	7	0	0

*Source*: Wong EB, Omar T, Setlhako GJ, et al. Causes of death on antiretroviral therapy: A post-mortem study from South Africa. PLoS One. 2012;7(10):e47542. https://doi.org/10.1371/journal.pone.0047542

ART, antiretroviral therapy; IRIS, immune reconstitution inflammatory syndrome.

Note: Categorised by the duration of ART at the time of death. Pre-ART deaths occurred in patients who were HIV-positive and eligible for ART but had not yet received it (CD4 cell count < 200 cells/mm^3^) or those who had received < 7 days of ART. Early ART deaths occurred between 7 and 90 days of ART. Late ART deaths occurred after > 90 days of ART.

† , All causes of death (immediate and contributing) are included and each patient may have ≥ 1 cause of death.

‡ , Non-infectious organ dysfunction, that is, pulmonary embolism or end-stage renal disease.

§ , At least one cause of death was revealed only through the post-mortem investigation.

**TABLE 2:** Guidelines for the management of acute pain at the end of life.^38^

Type of pain and treatment	Initial dosage	Comment
**Mild-to-moderate pain**		
Acetaminophen (Paracetamol)	1000 mg orally or rectally 3–4 times a day	Do not exceed 4 g per day. Use with caution in the presence of liver disease, particularly in the elderly and with concomitant alcohol use.
Ibuprofen (NSAIDS)	800 mg orally 3–4 times a day	GIT upset and bleeds, ulceration; Avoid in renal failure and use with caution in the presence of liver disease
Codeine	30 mg with/without 325 mg acetaminophen orally every 3–4 h as needed	Do not exceed 360 mg per day. Note that constipation is usual. Provide advice regarding diet, exercise and the use of laxatives. Codeine can be addictive and thus should be used under supervision.
Oxycodone	5 mg with or without 325 mg acetaminophen, orally every 3–4 h as needed	If analgesia is inadequate with initial treatment, adjust the dosage to 10 mg orally every 3–4 h as needed. As it can be addictive, use under supervision.
**Moderate-to-severe pain in patients not already on opioids**		
Morphine	Oral: 2 mg – 4 mg every 30–60 min as needed IV: 2 mg – 5 mg every 15–30 min as needed	For both morphine and hydromorphone, if analgesia is inadequate with initial treatment, increase the bolus dose by 25% – 50% for moderate pain or by 50% – 100% for severe pain. Morphine can be addictive and thus should be used under supervision.
Hydromorphone	Oral: 2 mg – 4 mg every 30 min as needed IV: 0.4 mg – 0.8 mg every 15–30 min as required	If the level of analgesia is acceptable, administer continuous infusion (equal to the total daily opioid dose) over 24 h, with a breakthrough dose every hour equivalent to 10% – 20% of the total 24 h opioid dose. If the current drug causes unacceptable side effects, administer an equianalgesic dose of a different opioid. As it can be addictive, use under supervision.
**Moderate-to-severe pain in patients already on opioids**		
Morphine/Hydromorphone	Bolus dose, up to 10% – 20% of total opioid taken in the previous 24 h, every 15–60 min as needed	If previously satisfactory analgesia becomes inadequate, increase the basal and bolus dose by 25% – 50% for moderate pain and by 50% – 100% for severe pain. For daily follow-up, calculate the total 24 h dose received, i.e., basal + breakthrough, and adjust the basal rate to equal this 24 h opioid amount; Adjust the bolus dose to 10% – 20% of this 24 h total. If the current drug causes unacceptable side effects, administer an equianalgesic dose of a different opioid.
**Neuropathic pain**		
Opioids	Adjust dose until analgesia has been achieved	-
Glucocorticoids	For example, 4 mg – 16 mg dexamethasone IV daily	Consider especially for acute neurologic injury such as nerve or spinal cord compression from a tumour.
Transdermal lidocaine patches	-	Consider especially when allodynia is present.
Short-acting antiepileptic drug, for example, gabapentin, pregabalin or tricyclic antidepressant	-	If survival of more than a few days is anticipated, consider adding one of these agents immediately.

*Source*: Blinderman CD, Billings JA. Comfort care for patients dying in the hospital. N Eng J Med. 2015;373:2549–2561. https://doi.org/10.1056/NEJMra1411746

NSAIDS, non-steroidal anti-inflammatory drugs.

However, at this time, oncology and radiotherapy on the African continent face multiple challenges: poor and ageing infrastructure, insufficient numbers of skilled professionals, inconsistent data collection, the huge rural–urban divide, empty drug shelves and constant drug stockouts, et cetera. This section briefly reviews this subject. Answers to the palliation of HIV-related cancer include greater access to prevention, for example, vaccination against the Human Papilloma Virus (HPV) and the broader reach of cervical health assessments in women, vaccination against Hepatitis B and the improved early diagnosis of cancer and the rapid referral to cancer curative treatment services. Closer collaboration between Africa’s oncologists, radiotherapists and HIV clinicians is needed if any of this is to be achieved.

**TABLE 3:** HIV+ concurrent disease and commentary.

Disease	Commentary
Cerebrovascular disease: Cerebrovascular accident	Comorbid disease, for example, hypertension, diabetes mellitus, renal disease, cardiovascular disease. Hyperlipidaemia with AZT, ddI, d4T and the PIs
Depression	May present with dementia, psychosis
Intoxication and medication	Toxicology screen: Alcohol and recreational drugs, efavirenz (EFV) encephalopathy and isoniazid (INH) encephalopathy. Steroid psychosis.
Progressive multifocal leukoencephalopathy (PML)	MRI = white matter lesions. IRIS following ART initiation = gadolinium enhancement on MRI
Metabolic encephalopathy	Vitamin B12 and folate deficiency, end-organ failure, antimicrobial encephalopathy, for example, metronidazole
Syphilis	Check the blood and CSF syphilis serological tests: VDRL, TPHA
Toxoplasma encephalitis	CT and MRI brain scan: Contrast (ring) enhancing lesions; antibody test positive in serum and CSF
HIV encephalitis: Untreated infection	CT and MRI: Loss of brain volume, prominent sulci, dilated ventricles, high CSF viral load. NB. Increased cells (lymphocytes) and marginally raised total CSF protein, with a normal glucose and negative tests for specific pathogens may be pointing to HIV itself as the cause of the encephalopathy.
HIV encephalitis: Viral escape or compartmentalisation syndrome^55^	CT and MRI as above. HIV viral load in CSF higher than serum viral load. CSF = active (cells, raised protein)
CMV encephalitis	PCR (viral load) and pp65 antigen in CSF, CMV retinitis and/or ulceration of the gastrointestinal tract (mouth to anus) may indicate ‘active’ CMV.
Bacterial meningitis, for example, TB meningitis (TBM)^56,57^	CSF = high protein, low sugar, cells (lymphocytes), TB found elsewhere, for example, LAM test (Urine) or gene XPert positive on sputum or CSF; TB culture (blood).
Fungal meningitis, for example, Cryptococcal meningitis (CCM)^58,59^	CSF = high protein, low sugar, cells, CrAg or CLAT positive yeasts seen, crypto culture on CSF positive.
Herpes simplex and varicella encephalitis	CT and MRI: Focal infarct or bleed, vasculitis on magnetic resonance imaging and angiography (MRA) to confirm a vasculitis, PCR (viral load) on CSF. Active shingles on the face, for example, ophthalmic division of the Trigeminal nerve, the Ramsay Hunt Syndrome.

AZT, Azidothymidine; MRI, magnetic resonance imaging; IRIS, immune reconstitution inflammatory syndrome; CSF, cerebrospinal fluid; VDRL, Venereal Diseases Research Laboratory; TPHA, Treponema pallidum haemagluttinin test; CMV, Cytomegalovirus; PCR, polymerase chain-reaction.

## Medical cannabis and palliative care for the HIV-infected people

The science of cannabinoid use as an adjuvant in HIV-related palliative care is new. Although South Africa has recently legalised cannabis for medical use, supporting data are largely observational and subjective. Table 3 summarises the current knowledge of cannabinoids in the control of common symptoms, for example, nausea, vomiting, pain, anorexia, insomnia, anxiety and depression. However, numerous ‘unknowns’ remain, for example, drug interactions and the dosing of a variety of HIV conditions and contexts. This is still a work in progress that will require updating in subsequent guidelines.

## Introduction

### What is palliative care?

**Definition 1.** According to the National Policy Framework and Strategy Policy on Palliative Care, Department of Health, South Africa, 2017–2022:

Palliative care is a multidisciplinary approach to the holistic care and support of patients and families facing a life-threatening illness. Its aim is to improve quality of life while maintaining dignity from diagnosis to death. For children, the spectrum of illness includes life-limiting conditions that may progress to death or may be severely disabling. Palliative care should be available to all patients *as* needed, *from birth until death,* and should be accessible at all levels of the health care service. Palliative care cuts across all health programmes in the delivery of services.^1^ The care of the dying is as old as the practice of medicine itself (see Box 1).

BOX 1 What is medicine?^79^

First I will define what I conceive medicine to be. In general terms, it is to do away with the sufferings of the sick, to lessen the violence of their diseases, and to refuse to treat those who are overmastered by their disease, realizing that in such cases medicine is powerless. (Hippocrates, c. 460–370 BCE)

*Source*: Shaner DM. Suspending ethical medical practice. N Engl J Med. 2010;363:1988

**Definition 2.** According to the World Health Organization (WHO):

Palliative care is an approach that improves the quality of life of patients and their families facing the problem associated with life-threatening illness, through the prevention, treatment and relief of suffering by means of early identification and impeccable assessment and treatment of pain and other problems, physical, psychosocial or spiritual. Palliative care:

- Affirms life and regards dying as a normal process.
- Neither hastens nor postpones death.
- Provides relief from pain and other distressing symptoms.
- Integrates the psychological and spiritual aspects of patient care.
- Offers a support system to help patients live as actively as possible until death.
- Offers a support system to help the family cope during the patient’s illness and during the experience of bereavement.^2^

**Definition 3.** End-of-life care: This refers to healthcare, not only of a person in the final hours and days of his or her life, but more broadly care of those with a terminal condition that has become progressive and incurable (Wikipedia, accessed 07 October 2018). The National Council for Palliative Care, United Kingdom, states that end-of-life care is given to persons ‘likely to die within 12 months’. Several commentators make the point that end-of-life care is not identical to palliative care, that is, end-of-life care is a final phase within the broader provision of palliative care. Nonetheless, its starting point is sometimes difficult to identify.

**The principal goal of palliative care is the relief of suffering**. This is done through individualising care – addressing symptoms, controlling pain, listening to the patient, responding to fear and anxiety, incorporating families and the patient within a competent, professional and resourceful team, and bereavement support for the patient’s loved ones. A defining characteristic of palliative care is the notion of *total pain*, the recognition that pain cannot be alleviated completely unless all contributing factors that inform the patient’s life experience are addressed. Palliative care is *team work*. The team comprises a range of medical, nursing, paramedical and psychosocially trained health and community workers. The leader is usually a senior palliative care-trained clinician. The South African National Policy Framework on Palliative Care envisages that these teams will be accessed by patients – including those who are human immunodeficiency virus (HIV)-positive – at the community, district and regional levels in free-standing clinics and at all district, secondary and tertiary-level hospitals across the country. These proposals are currently aspirational, however, as significant barriers to implementation still exist: approval and financing, recruitment and staffing, education and training, et cetera.

**The individual matters**. The process of palliative care is just, non-judgmental and given to all irrespective of colour, creed or class. ’The relief of suffering is considered one of the primary ends of medicine by patients and the general public’.^3^

Yet patients and their families do not necessarily agree with health workers on the means to this end.

‘In the care of the dying, patients and their friends and families do not divide suffering into its physical and nonphysical sources the way doctors, who are primarily concerned with the physical, do.’^3^

Palliative care recognises this divide and addresses it by taking all causes of suffering into account when aligning the goals of the patient with that of the caregiver and healthcare system. A consultative, multi-disciplinary approach forms the basis of this model of care.

## The HIV epidemic in South Africa in 2018, palliative care: Defining the need

### HIV morbidity and mortality

The Joint United Nations Programme on HIV and AIDS (UNAIDS) reported in 2016 that 7.1 million South Africans were HIV-positive.^4^ It was estimated that 56% were on antiretroviral therapy (ART) yet viral control could only be confirmed in 45%. Although 95% of pregnant women had accessed ART, mother-to-child transmission of HIV in 2016 still resulted in 12 000 newborn infections. The 2017/2018 SA Human Sciences Research Council’s (HSRC) report, released in July 2018, estimated that 7.9 million South Africans are now living with HIV, an increase of 1.6 million over the past 5 years; 70.6% are taking ART; and 87.5% of PLWHIV aged 15–64 years on ART have suppressed viral loads. The number of new cases among women aged 15–24 years has remained high since 2012 and is three times that of their male peers, while the overall prevalence of HIV among South Africans was 14%. Prevalence rates were higher in the provinces of KwaZulu-Natal (KZN), Mpumalanga (MP), the Free State (FS) and the Eastern Cape (EC), with rates of 18.1%, 17.3%, 17.0% and 15.3%, respectively.^5^ Notwithstanding these numbers, new HIV infections have fallen by 44% since 2012 and HIV testing has been on the rise.

The evolution of the South African epidemic has meant that many who are infected yet undiagnosed and ART naïve, or who have defaulted on ART in the past, now present to hospitals and clinics with symptomatic disease, namely, the HIV-sick. It is estimated that as many as 40% – 60% of the beds in the public hospitals in KZN, Gauteng, MP and FS are occupied by PLWHIV.^6,7^

Few, if any, state hospitals offer a formal palliative care service and despite access to ART since 2005, mortality from HIV-related diseases remains high. Of the 14 431 patients who died in Soweto’s Chris Hani Baragwanath Hospital from 2006 to 2009, 64% of the men and 82% of the women were HIV infected. More than 90% of those dying between the ages of 30–40 years were HIV infected.^8^

### Causes of death of hospitalised HIV-sick in South Africa

A small but well-analysed autopsy group from the Charlotte Maxeke Johannesburg Academic Hospital (CMJAH) is reported in Table 1.^9^ The dominant regional pathogen of the HIV-infected patients is *Mycobacterium tuberculosis.* All who died of TB had disseminated infection at the time of their death. The first 90 days following the start of ART, namely, ‘early-ART’, is a key risk period for death from TB. The recovering immune system has been implicated in these deaths, namely, the immune reconstitution inflammatory syndrome (IRIS) as the likely cause or complicating event in these deaths.^10^

**FIGURE 2:**
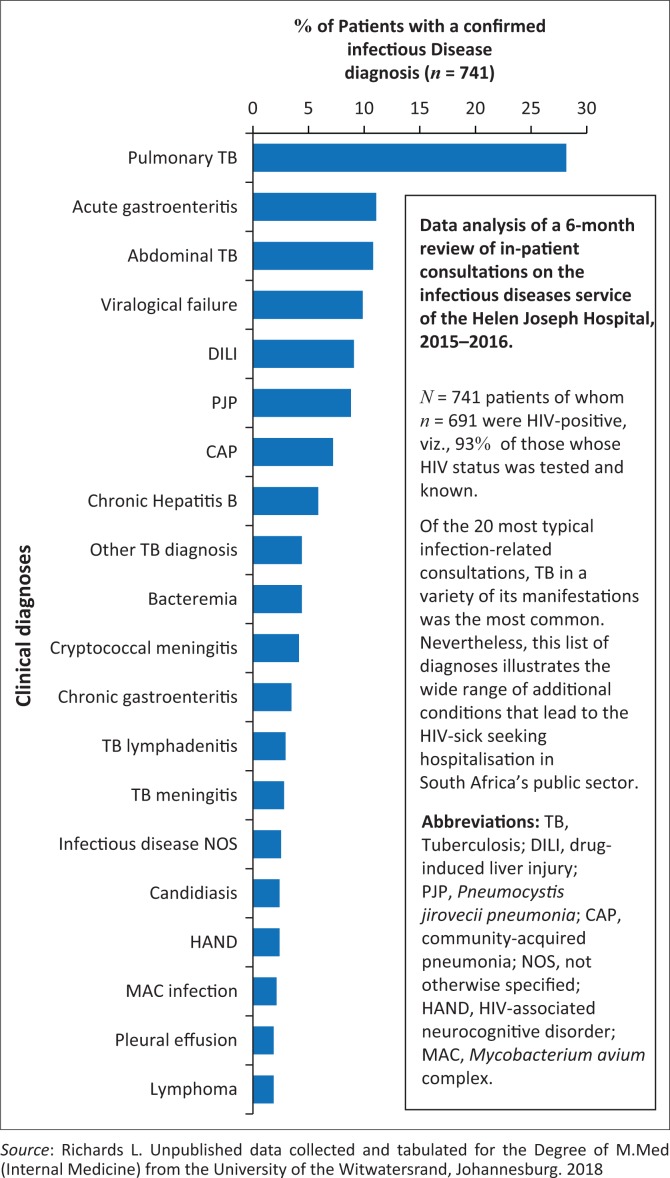
Medical diagnoses of a 6-month review of in-patient infectious disease consultations at the Helen Joseph Hospital, Johannesburg, 2015–2016.^11^

Figure 2 summarises the admission diagnoses of 741 patients evaluated during a 6-month period on the Infectious Diseases consultation service of the Helen Joseph Hospital, a public hospital in Johannesburg, of which 93% of the consulted patients were HIV-infected. The individual bars demonstrate the wide variety of conditions that affect the acutely sick. Although TB is the most frequent diagnosis, it is one of many life-threatening conditions in this group of patients. Note that virtually all these conditions are treatable and the majority can be cured.^11^

## Models of HIV-palliative care: Addressing the need

Models of palliative care vary. Three models that are frequently encountered by the HIV clinicians in South Africa (SA) are briefly discussed in this section.

### No formal palliative care provided

**FIGURE 3:**
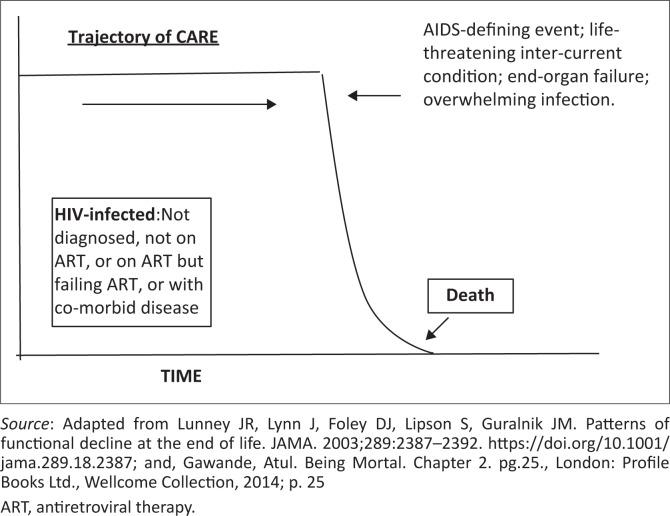
The HIV-infected patient, no palliative care.^12,13^

In this model (Figure 3), little or no palliative care is accessed prior to death. The HIV infection is not well controlled or is undiagnosed prior to admission, although the patient has been admitted to hospital acutely ill. Although the risk of death is often high, palliative care is not offered nor is it integrated with acute care. Suffering is not adequately relieved and patients and their families often feel abandoned by the public healthcare system. This model of care is the usual experience of the HIV-sick at this time in SA (for additional comments, see Appendix 1).

### Intermittent palliative care given during periods of need: The HIV-infected patient on antiretroviral therapy with long-term viral control and immune reconstitution

**FIGURE 4:**
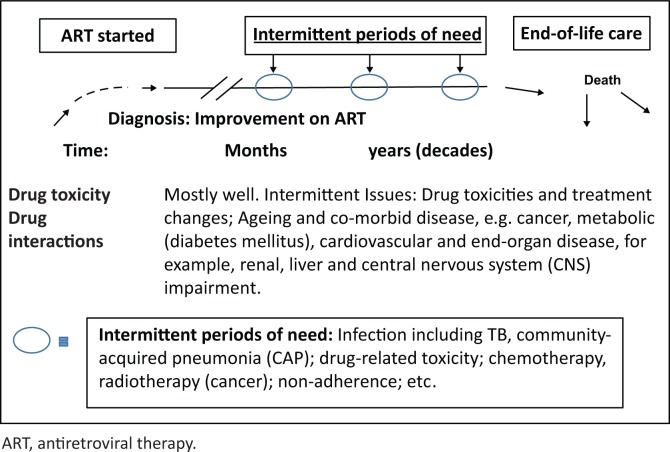
Palliative care during periods of need.

In this model, the patient on ART initially improves but requires assistance to cope with drug toxicities and possible drug interactions. Over time, the need for both acute curative and palliative care is encountered during periods of serious comorbid disease, failure of ART, re-introduction of active ART and, finally, during special health needs towards the end of life^14^ (see Figure 4). Illness increases in importance and frequency with the ageing of HIV survivors as does its complexity and costs.

### Integrated palliative care for people with progressive co-morbid disease

**FIGURE 5:**
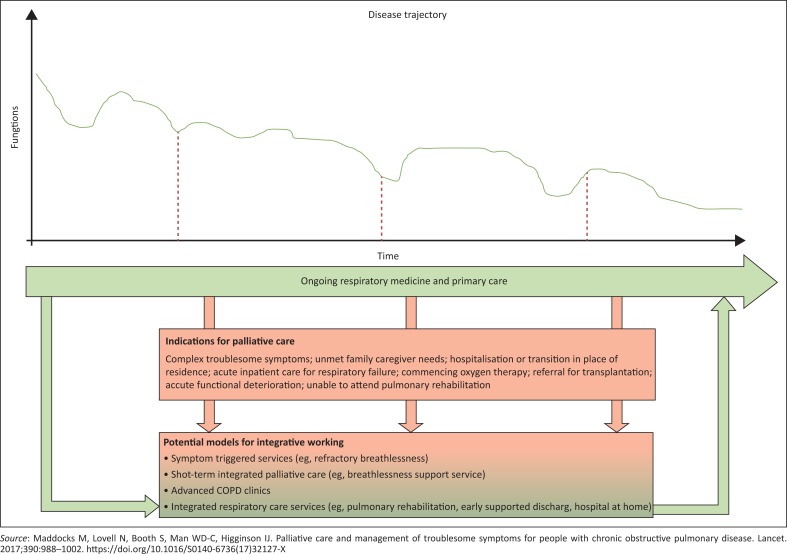
Integrated palliative care for people with progressive co-morbid disease, for example, chronic obstructive pulmonary disease (COPD).^19^

Chronic lung disease (CLD) is a problem for long-term survivors of HIV-infection.^15,16^ Infection from birth, inadequate or intermittent viral control and inter-current respiratory tract infections characterise a group of adolescents with advanced and irreversible small airways disease.^17,18^ Chronic lung disease also affects HIV-infected adults on ART, many of whom are smokers, often men and frequently domicile in high-income countries.^19^ In this model of HIV, people with an active comorbid condition, such as chronic obstructive pulmonary disease (COPD), chronic renal failure, heart disease et cetera, experience a slow but progressive downhill trajectory. Each new episode of disease or hospital admission compromises organ function further and moves the patient towards the end of life.^20^ The model in Figure 5 also applies to other chronic diseases experienced by the HIV-infected, namely, non-AIDS-defining cancers, progressive neurocognitive impairment, autoimmune conditions and bone and joint diseases.

### The traditional palliative care model: From diagnosis to bereavement

**FIGURE 6:**
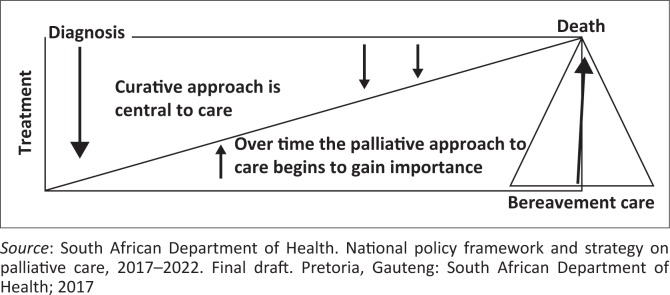
Current concept of palliative care: From diagnosis to bereavement.^1^

Palliative care is a continuum of care that starts from the time of diagnosis of a life-threatening illness or condition (e.g. AIDS) and continues until a time after death when bereavement support is provided to the family members of the deceased.^1^ During this period, emphasis gradually moves from the curative to the palliative, as dictated by the changing needs of the patient and family (see Figure 6).

Patients on ART who regain normal or near-normal immune activity and whose viral count is ‘undetectable’ (i.e. viral load [VL] < 40–50 copies/mL) have life expectancies similar to that of their uninfected peers.^21^ While the availability of ART has brought great benefits, many still unfortunately die. Under normal circumstances, the clinician’s mandate is the restoration of a patient’s health and well-being which begins with obtaining a medical history, the examination of the patient, the formulation of a probable diagnosis, investigation, the start of remedial therapy, the monitoring of the response, a review of laboratory or radiographic data and the confirmation of the diagnosis. Patients want and need to feel better and good symptom control is the shared ground of curative and palliative medicine. It addresses the immediate needs of those who suffer. A recent randomised controlled trial (RCT) of palliative care in patients with advanced non-small-cell lung cancer found that this intervention reduced depression, improved quality of life and produced a short but significant 3-month survival advantage.^22^

## Eligibility for home and hospice support: Assessing the need

### Comment

For most people, ART preserves and restores immune function and extends survival. However, in every HIV clinic, a small group of patients fail to reconstitute CD4 cells despite ART and viral control. These ‘immune non-responders’ (INRs) are often older (> 45 years), start ART late, begin ART at a very low CD4 nadir/baseline (viz. < 100 cells/mm^3^) and give past medical histories that suggest advanced immune suppression, namely, previous TB, recurrent bacterial and other infections and unexplained weight loss.^23^ Despite adherence to therapy, INRs remain at an increased risk of disease and death notwithstanding years of documented viral suppression.^24^ No dependable treatment currently reconstitutes the blood compartment with CD4 cells in this group of patients. Attempts to address this situation by changing background ART in those who are virologically suppressed have not been successful and this is mainly discouraged. Where the patient’s usual medication may be incriminated, for example, AZT or trimethoprim-sulfamethoxazole, alternative drugs should be tried.

*Prevention*, such as commencing ART as soon as possible after the start of HIV infection when CD4 cells are likely to be abundant and the thymus is functional, is the therapeutic safeguard against immune non-response to ART.^25^

In addition to this group are those HIV-infected with identifiable causes of immune suppression, for example, steroid use, alcohol abuse, herbs such as the ‘African potato’, chemotherapy, infections such as TB and *Mycobacterium avium* complex (MAC), malignancies and rheumatological conditions such as systemic lupus erythematosus (SLE), rheumatoid arthritis and drugs that are used to control these conditions.

Management of the comorbid condition or removal of the offending toxin, for example, alcohol or steroids, may improve the immune response of these patients. The likelihood of cannabinoid use influencing the human immune system either negatively or positively is currently unknown. If these agents are indicated for the palliation of symptoms, it seems reasonable to continue to monitor CD4 response as per the usual intervals recommended in current ART guidelines.

### Morbidity and mortality risk from HIV infection: General indicators

While all should be able to access the costlier components of a palliative care service, for example, admission to hospice and home nursing, it is acknowledged that the capacity of palliative care services in SA is limited. Who are in greatest need? No randomised, controlled palliative care trial addresses this question directly. Nevertheless, several observational studies have identified associations that provide some answers (see Appendix 2); these indicators change and their significance may diminish or reverse with time on ART. The following baseline characteristics in a large South African observational study influenced mortality in the first year after starting ART: WHO stage, body mass index (BMI), haemoglobin level, CD4 cell count, HIV plasma VL and symptoms. Yet, once on long-term ART, only CD4 cell count, BMI, haemoglobin level and a suppressed VL retained survival significance.^26^ Another observational study that included data from South, Central and West Africa found that age, gender and baseline CD4 cell count continued to predict death at 6, 12, 24 months and beyond while taking ART.^27^ These factors indicate the need of palliative care but are not to be used to define ‘therapeutic futility’, that is, the discontinuation of active care. In every circumstance, the patient must be treated with dignity and respect and every attempt made to correct the underlying life-threatening process and to offer where possible, life.

### Mortality indicators: Assessment tools

#### Karnofsky score

Although scores of ≤ 50% are often used as a qualifier for hospice admission, scores of < 60% are significant and should trigger consultation with the palliative care team.^28^ However, given the potential for ART to reverse underlying disease in the HIV-infected – the Lazarus effect – the absolute score must be viewed with caution. The usefulness of the Karnofsky score as a measure of impending mortality in this group of patients has not been established (see Appendix 3 for more details).

#### The Support and Palliative Care Tools

The Support and Palliative Care Tools (SPICT^TM^) are used in the United Kingdom and have been applied to patients with HIV and cancer.^29^ The tool is not specific to HIV and does not consider the role of ART and the curative potential of the treatment in several life-threatening infections, for example, sulfonamides in cerebral toxoplasmosis and *Pneumocystis jiroveci* pneumonia (PJP), TB drugs in disseminated TB, antifungals in cryptococcal meningitis (CM), et cetera.

- **General indicators:**
  ■ Increasing physical and/or mental regression or dependency
  ■ A low BMI (viz. < 18 kg/m^2^ and/or 5% – 10% loss of body weight)
  ■ Ongoing and troublesome symptoms
  ■ Plea for supportive and palliative care or the wish to die.

**Assessment:** Two or more ‘general’ indicators = unmet supportive and palliative care needs = qualify for palliative care benefits.

- **Clinical indicators:**
  ■ Worsening frailty and cognitive/neurological, cardiovascular and/or respiratory status
  ■ Worsening of cancer
  ■ Onset of life-threatening end-organ failure.

**Assessment:** One or more ‘clinical’ indicators = unmet supportive and palliative care needs = qualify for palliative care benefits (see Appendix 5 for more details).

#### The Veterans Aging Cohort Study index

This scoring system (Veterans Aging Cohort Study – VACS index)^30^ was developed in North America and Europe, focuses on the HIV-infected and uses seven clinical or laboratory indices that assist with the long-term (viz. 6 years) prediction of survival (death) following the start of ART. Although the data reflect the experience of high-income countries and examine the risk of death in patients already on ART, the database is large, the index has wide applicability and its predictive value is superior to individual and composite indices currently in use.^31,32^ Most of the indices used in this system, with the exception of baseline hepatitis C virus (HCV) serology, are routinely performed in the public sector in SA. Local HCV prevalence is low (viz. 3% – 5%) and the absence of this index in the calculation of the score is unlikely to influence the result (see Appendix 6 for more details).

#### Choosing an assessment tool for South African patients

According to Merlin et al.,^33^ ‘[*a*]ny attempt at prognostication that does not address whether the patient has had an adequate trial of ART would be ill-informed and inaccurate’.

In the United States, an estimated life expectancy of ≤ 6 months is a sufficient reason to access hospice support,^33^ although no similar timeline has been formally adopted in SA. While the VACS index has definite merit, it was not intended to answer the question of eligibility to specialised hospice benefits, namely, home care and hospice admission. The SPICT tool has wide applicability but fails to take in the reversal of clinical disease on ART. This committee recommends the use of the SPICT tool where ART is unlikely to improve outcome, for example, end-stage cancer and irreversible end-organ failure, or where current or future ART is judged by the medical and palliative care team to be futile. With regard to patients in SA’s private sector, provision of palliative care and hospice benefits is addressed in and supported by the *South African Medical Schemes Act* 131 of 1998.^34,35^ At this time, it is recommended that Medical Schemes utilise the SPICT tool when considering the reward of hospice benefits and/or the ≤ 6 month estimated survival time as practised in the United States. However, the patient must be on ART or switch to active ART if treatment failure has been confirmed and where ART remains a therapeutic option. Every HIV-infected person should be given the opportunity to access ART and enjoy its benefits. Where a patient refuses to take ART, yet requires hospice admission, addressing the patient’s immediate need is of primary importance and the question of starting ART can be postponed.

## Managing the HIV-sick: Symptom control

### Pain control (see Box 2, Table 2 following, and Table 3-A6 in Appendix 6).

Of all treatment modalities reviewed, the best evidence for pain reduction averages roughly 30% in about half of treated patients, and these pain reductions do not always occur with concurrent improvement in function. These results suggest that none of the most commonly prescribed treatment regimens are, by themselves, sufficient to eliminate pain and have a major effect on physical and emotional function in most patients with chronic pain. (Turck et al.^36^, p. 2232)

BOX 2 The pain assessment chart: Key objectives.^36^

**Objectives in the assessment of severe pain**

1. **Characterise the multiple dimensions of the pain:**
  - Intensity
  - Temporal features: onset, course, daily fluctuation and breakthrough pain
  - Location and radiation
  - Quality
  - Provocative or relieving factors
2. **Formulate an understanding of the nature of the pain:**
  - Cause
  - Probable pathophysiology
  - The pain syndrome: neuropathy, cancer pain, post-herpetic neuralgia, et cetera.
3. **Characterise the effect of the pain on quality-of-life domains:**
  - Effect on physical function and well-being
  - Effect on mood, coping and related aspects of psychological well-being
  - Effect on role functioning and social/familial relationships
  - Effect on sleep, mood, vitality and sexual function
4. **Clarify the ext ent of concurrent disease and review the planned treatment and prognosis.**
5. **Clarify the nature and quality of previous laboratory tests and previous treatments.**
6. **Review the HIV and ART history:**
  - Approximate duration of HIV infection and manner of confirmation
  - Counselling and disclosure
  - Most recent CD4 count and viral load, plus all available lab results, including FBC, U&E, LFTs and HBV/HCV status
  - ART exposure, adherence and viral failure/drug resistance
  - Prior opportunistic infections, for example, TB, PJP, CCM and malignancies
  - Current symptoms in addition to pain.
7. **medicalElucidate medical comorbidities.**
8. **Elucidate psychiatric comorbidities:**
  - Substance-use history
  - Depression and anxiety disorders
  - Personality disorders
9. **Identify additional needs that require palliative care interventions:**
  - Additional symptoms
  - Vulnerable/at-risk populations: children, specific sexual identity groups, for example, sex workers, injection drug users, migrants and refugees
  - Distress related to psychological or spiritual concerns
  - Caregiver burden and concrete needs
  - Problems in communication, care coordination and goal setting

*Source*: Turck DC, Wilson HD, Cahana A. Treatment of chronic non-cancer pain. Lancet. 2011;377:2226–2235. https://doi.org/10.1016/S0140-6736(11)60402-9

ART, antiretroviral therapy; FBC, full blood count; U&E, urea and electrolytes; LFT, liver function test; HBV, hepatitis B virus; HCV, hepatitis c virus; TB, tuberculosis; PJP, *Pneumocystis jirovecii* pneumonia; CCM, Cryptococcal meningitis.

Chronic pain, that is, pain lasting ≥ 3 months, is common in the HIV-infected population and is often the reason for a palliative care consultation.^31^ Pain in HIV-infected South Africans is likely to accompany an identifiable clinical cause and the most valuable contribution the HIV and palliative care physician can make to pain management is to identify the cause and treat it. The severity of the pain must be documented: ask the patient to describe the pain and to rate it on a scale of 0 = ‘no pain’ to 10 = ‘the worst pain I have ever experienced’. Can the pain be fitted into a recognisable pattern, for example, peripheral neuropathy, post-herpetic neuralgia and meningeal irritation, or is it associated with a specific site or organ, for example, perianal ulcers and bedsores, pulmonary or pleural disease, a tumour such as Kaposi’s sarcoma or a collection of enlarged lymph nodes? While the clinical examination will provide some answers, a pain assessment chart will ensure that no aspect of the pain is omitted (see Box 3).

BOX 3 Thomas Browne, *The Religio Medici*.^75^

Men that look no further than their outsides, think health an appurtenance unto life and quarrel with their constitutions for being sick; But I that have examined the parts of man, and know upon what tender filaments that fabric hangs, do wonder that we are not always so; And considering the thousand doors that lead to death, do thank my God that we can die but once.

– Thomas Browne, 1642. *The Religio Medici*

*Source*: Ferry G. Thomas Browne: A rarity among rarities. Lancet. 2017;389:1687–1688. https://doi.org/10.1016/S0140-6736(17)31067-X

The control of pain is a priority and must be addressed as soon as the patient arrives at a clinic or the admission ward. Tests to confirm the diagnosis are important but must not be allowed to delay the treatment of the pain, which works best when given by an interdisciplinary team.^36^ Ideally, the team should offer, in addition to good medical and nursing care, most or all of the following:

- physical rehabilitation
- exercise therapy
- cognitive restructuring that emphasises self-management, self-efficacy and resourcefulness, that is, activity rather than passivity, reactivity, dependency and hopelessness
- behavioural treatment, for example, relaxation and/or engagement in activities that enhance functionality
- vocational rehabilitation if long-term survival is not in doubt
- drug management – in particular, the avoidance or reduction of opioid dependency. *Nota bene* evidence-based support for opioids as improvers of chronic pain and functionality is weak; therefore, their use must be measured against their risks, for example, addiction, hypogonadism, falls and fractures, depression, overdose and death.^36^

It is common to treat pain in a stepwise manner, starting with non-opioid medication such as paracetamol (acetaminophen), aspirin and the non-steroidal anti-inflammatory drugs (NSAIDs) and ending with the opioids or combinations of opioids and non-opioids^37,38^ (Table 2).

The 2017 Chronic Pain Guideline of the HIV Medicine Association of the Infectious Diseases Society of America (IDSA) provides an evidence-based evaluation of pain and its management in the HIV-infected people.^39^ In this assessment, only the following aspects of pain care received unanimous – ‘strong quality, high level’ – support:

- Any ‘new pain’ in a patient with previously controlled pain needs fresh re-assessment.
- Acetaminophen and NSAIDs are the first-line agents for the treatment of musculoskeletal pain in persons living with HIV.
- Topical capsaicin is indicated for chronic HIV-associated peripheral neuropathy in conjunction with additional analgesics and supportive therapies and adequate viral control.
- Screen all with chronic pain syndromes for depression, that is, direct questioning, via a depression questionnaire and/or through a psychiatric referral.

Additional remarks that scored well (viz. for their strong quality of evidence) but did not secure unanimous support from reviewers included:

- The re-evaluation of pain among those with a ‘changing experience of pain’.
- The ‘immediate or early’commencement of ART in those with a sensory polyneuropathy believed to be caused by HIV infection.
- Gabapentin, with dose escalation up to a maximum of 2400 mg daily po in divided doses, is recommended as the first-line oral treatment of chronic HIV-associated neuropathic pain (the authors note that somnolence occurs in 80% and can be problematic). The serotonin-noradrenaline reuptake inhibitors, tricyclic antidepressants and pregabalin received only weak or moderate support.
- Despite the absence of RCTs in HIV-related pain syndromes, Alpha-lipoic acid (ALA) received support for neuropathic pain treatment in view of its confirmed role in diabetic neuropathy, a condition that also targets the peripheral nerves.
- Opioid analgesics are not indicated for first-line control of neuropathic pain or chronic pain syndromes. Tramadol, a combination opioid, that is, a serotonin + noradrenaline reuptake inhibitor + a µ-opioid agonist, received support, however, for use in several non-cancer pain syndromes, including osteoarthritis, fibromyalgia and the neuropathic pain syndromes.^36^

#### Opioid therapy

*Opioid therapy* is often the treatment of choice for patients with cancer and those having moderate to severe pain.^38,39^ Although the WHO analgesic ladder recommends different opioids for different levels of pain, for example, codeine for moderate pain and morphine for severe pain, cautious dose escalation using the initial opioid may avoid an unnecessary switch.^38,40^ Morphine can be given by several routes and subcutaneous, intramuscular and intravenous injection should be considered for those unable to swallow. Fentanyl is given by injection and transdermal skin patch but is not widely available to public sector patients in SA. Unwelcome respiratory complications can be avoided with careful monitoring of opioid dose increases, which is particularly important where patients and families have expressed a desire for a pain-free yet lucid end to life.

Drug–drug interactions are important to note. Many drugs used in pain control, for example, opioids, benzodiazepines, antidepressants and sedatives, are substrates of the cytochrome P450 (CYP450) family of enzymes. Strong inducers or inhibitors of CYP450 will reduce (inducers) or potentiate (inhibitors) serum levels (efficacy and toxicity) of these drugs. Several antiretrovirals (ARVs) – especially the non-nucleoside reverse transcriptase inhibitors (NNRTIs – usually inducers), protease inhibitors (PIs – inhibitors) and drugs commonly used in the management of the HIV-sick, for example, rifampicin (inducer) for TB, carbamazepine and phenytoin sodium (inducers) for seizures, will influence the activity of substrates of this enzyme pathway (see Appendix 6, Table 2-A6 and Table 3-A6).

The non-medical use of prescription opioids, which has become a major public health issue in the United States and Europe,^41^ should not be withheld from people in need in low- and middle-income countries (LMICs), for example, Africa and Asia. The authors of a recent Lancet Commission on Palliative Care and Pain Relief provide data to show that this happens:

The fact that access to such an inexpensive, essential and effective intervention – opioid use for pain relief – is denied to most patients in LMICs and in particular to poor people – including many poor or otherwise vulnerable people in high-income countries – is a medical, public health and moral failing and a travesty of justice.^42^ (p. 1391)

The Commission notes that the 10 health conditions that result in the largest numbers of patients seeking palliative care in LMICs, namely, malignancy, cerebrovascular disease, lung disease, injuries, TB, premature birth, HIV, liver disease, non-ischaemic heart disease and dementia, also account for about 95% of days of serious health-related suffering reported in these countries.

### Fatigue, weakness, anorexia and wasting (see Table 3-A4, Appendix 4)

Increased resting energy expenditure (REE) is a persistent accompaniment of HIV infection despite the use of ART.^43^ In situations of inadequate food intake and altered food metabolism, weight loss may become severe. With every 1% increase in unintentional weight loss from the baseline measurement, there is a detectable increase in the risk of death of the HIV-infected patient.^44^ The lower the BMI, that is, below the normal value (BMI 18 kg/m^2^ – 20 kg/m^2^), the greater the risk.^45^

Possible clinical conditions include:

- Disease of the mouth and upper gastrointestinal tract (GIT), for example, **oesophageal candidiasis** and **cytomegalovirus (CMV)** infection and ulceration.
- Anorexia. Consider medication, tumour and occult infection, for example, **TB** (drug-resistant TB) or MAC. Drugs: **Chemotherapy, lopinavir/ritonavir**.
- Food insecurity. Particularly among refugees, migrants and the poor.
- Malabsorption, for example, chronic diarrhoea, **GIT-TB or MAC**, tumour of the GIT. Send repeated stool samples (× 3) for parasites: ***Cryptosporidia, Cystoisospora* spp**. Poorly controlled HIV infection = HIV enteropathy.
- Infection, particularly **end-stage HIV, TB, MAC** and **CCM**.
- Disseminated **tumour**.
- Hormonal deficiencies, for example, Addison’s disease, hypothyroidism and hypogonadism.

**(Bold** indicates conditions that are frequent among the HIV-infected patients.)

**Management**: The HIV clinician will look for treatable causes but as the patient approaches death, symptomatic treatment will take priority. Start ART if the patient is treatment naive or check for resistance if on ART and the VL is detectable. Glucocorticoids, for example, dexamethasone 2 mg daily po or IV–4 mg daily po or IV, can be considered at the end of life as the risk of additional immunosuppression is unlikely to influence the outcome. Steroids are often given to stimulate the appetite and/or to counter the fatigue and weakness of the final illness. Vigilance is indicated if steroids are given for a prolonged period of time and patients should be monitored for hypertension, hyperglycaemia, gastrointestinal bleeds, fluid retention, proximal myopathy, confusion and psychosis. Testosterone has benefited hypogonadal individuals with fatigue^46^ and the stimulants, modafinil and armodafinil, have had success in reducing fatigue in small RCTs, but caution is advised in view of limited data and the risk of drug abuse.^31^ Invasive procedures, such as nasogastric feeding and percutaneous endoscopic gastrostomy, are discouraged at the end of life but may be considered where a reversible condition has been identified. Ensure good nursing, keep the oral mucosa clean, moist and wet and mobilise the indigent patient where possible. Avoid bedsores and treat oral thrush with topical nystatin drops 2 mL – 5 mL ‘swish and swallow’ five times a day or oral fluconazole 50 mg – 100 mg daily until the infection clears. If oesophageal candida is diagnosed, give oral fluconazole 100 mg – 200 mg daily for 7–10 days. A short course of IV amphotericin B can be considered if the patient is unable to swallow the fluconazole. Treat oral and oesophageal *Herpes simplex* aggressively, prescribe oral acyclovir (public sector) or valaciclovir (private) if the infection is mild and confined to the mouth and IV acyclovir for disease that is extensive, virulent or involves the oesophagus. Continue treatment until the infection has cleared, usually for 7–10 days, and control the pain that often requires strong analgesia, for example, codeine or tramadol. Food should be liquidised or very soft to minimise oral pain while the infection is acute.

### Dyspnoea and cough (see Table 3-A4, Appendix 4)

The anatomic connections that control the cough reflex link the upper and lower airways to afferent and efferent neural connections within the brain.^46^ Disease at any of these sites may be responsible for cough; however, in HIV-infected patients, cough usually directs attention to the lung. An acute cough lasts from a few days to a few weeks and a chronic cough lasts for ≥ 8 weeks.^47^

Symptoms of dyspnoea and cough include the following:

- **Bacterial pneumonia**: Productive cough, fever, chest pain, sudden onset and acute history. Aspiration pneumonia is frequent at the end of life.
- **Pulmonary TB** (PTB): Productive cough, haemoptysis, chest pain, fever, night sweats, weight loss, chronic history or past history of TB, and TB contact history.
- **PJP**: A dry incidental chronic cough, worsening dyspnoea with effort, later dyspnoea at rest, severe hypoxia at rest, worsens with exertion, little or no fever, minimal chest pain, profound fatigue and exhaustion, tachycardia and unrelenting tachypnoea. Often a bedside diagnosis!
  ■ **PJP**: **Poor prognosis**. The persistence of hypoxia or respiratory failure, tachypnoea and tachycardia despite treatment, no decrease in baseline LDH level with treatment, worsening of the chest X-ray (CXR) on treatment, elevated Acute Physiology and Chronic Health Evaluation (APACHE) II score.^48,49^
  ■ **PJP: Prevention**. Initiation onto ART soon after the initial infection with HIV and prophylactic use of trimethoprim-sulfamethoxazole with all baseline CD4 cell counts < 350 cells/mm^3^ are recommended.^50,51^
  ■ **PJP: Ethics.** The decision to admit to the intensive care unit (ICU) and to ventilate usually rests with the ICU clinician or resident pulmonologist; however, this action may be futile, that is, it does not guarantee success or survival. The palliative care team must make itself familiar with this scenario in order to provide wise support (insight and understanding) to patients and colleagues and, where possible the death of a patient in the ICU and on a ventilator should be avoided. This is a source of great suffering for patients and their loved ones, although it is best managed through discussion with the patient, family and friends before the urgent need of ICU admission and ventilation arises.

Causes of dyspnoea and cough include the following:^47^

- **Acute infection:** Trachea-bronchitis, **pneumonia** (viral and bacterial).
- **Chronic infection**: Bronchiectasis, chronic lung infection (bacterial, e.g., **TB**, **MAC**, lung abscess), fungi (**PJP, cryptococci**, histoplasmosis), protozoa (toxoplasmosis) and rarely helminths.
- Airway diseases: Asthma, chronic bronchitis, postnasal drip.
- Diseases of the parenchyma and vessels of the lung: Chronic interstitial lung disease, emphysema, sarcoidosis, pulmonary vascular disease, namely, embolic, vasculitis, pulmonary hypertension.
- **Tumour**: Primary and secondary tumours of the lung and chest cavity.
- Cardiovascular disease: Left ventricular failure, right-sided endocarditis.
- **Anaemia**: Severe dyspnoea, Hb < 8 g/dL.
- GIT: Acid reflux, aspiration pneumonia (cough often at night).
- Metabolic acidosis: Diabetic ketoacidosis, **lactic acidosis (NRTIs: zidovudine, stavudine), renal tubular acidosis (tenofovir disoproxil fumarate):** Tachypnoea and dyspnoea but with normal oxygenation (p0_2_), chest auscultation normal, CXR clear.
- Drugs: Angiotensin-converting enzyme (ACE) inhibitors (cough).

(Note: **Bold** indicates conditions that are frequent among the HIV-infected patients.)

**Management**: Many of the conditions that present with cough and dyspnoea in the HIV-infected patient are treatable. However, some conditions, such as non-responsive PJP or end-stage (destroyed) lung disease whether from previous TB or from COPD, may not be reversible and will require palliation. Blood gas measurements, blood transfusion, intubation and ventilation are likely to be inappropriate when death is imminent. Supportive care such as oxygen, bronchodilators, steroids and antimicrobials may be prescribed if indicated and diuretics may be needed to treat pulmonary oedema and fluid overload. **Opioids** are regarded as the treatment of choice for dyspnoea in the context of end-of-life care. These can be given safely, that is, without the risk of respiratory depression, if titrated carefully with attention to dose and response.^38^ In addition, the careful use of **benzodiazepines** may relieve anxiety associated with breathlessness and asphyxiation.^38^

Specific end-of-life therapies include the following:

- **Morphine:** 5 mg po– 10 mg po every 30 min as needed until the patient is comfortable, or IV 2 mg – 4 mg every 30 min to 1 h as needed until the patient is comfortable.
- **Oxygen**: Adjust to achieve satisfactory saturation and subjective relief of dyspnoea.
- **Non-pharmacologic measures**: Support, relaxation and breathing exercises, et cetera.

### Dry mouth

Causes of dry mouth include:

- Poor oral immunity: Often a sign of advanced HIV and AIDS with or without HIV-associated dementia (HAD); poorly controlled diabetes mellitus, gingivitis
- Infection: Chronic parotitis, salivary duct obstruction (stones, tumour), extensive **oral candidiasis**
- Autoimmune: Sjögrens syndrome
- Drugs: Anticholinergics, alpha- and beta-blockers, diuretics, calcium channel blockers
- Post-irradiation, for example, cancer of the head and neck.

(Note: **Bold** indicates conditions that are frequent among the HIV-infected patients.)

**Management:** Address the cause, clean the mouth regularly, maintain hydration – oral fluids if able to swallow, chewing gum and mildly acidic sweets (lemon drops), stop offending drugs (above), artificial saliva.

### Nausea and vomiting (N&V)

Nausea is defined as the subjective feeling associated with the action of vomiting. Identify and treat the cause, including the common associations mentioned below:

- Abdominal conditions: **Gastroenteritis**, intestinal diseases (inflammatory, obstructive)
- General causes: Middle ear and **CNS disease,** for example, **meningitis and raised intracranial pressure (RICP)**; Anxiety and fear; cardiac, for example, acute myocardial infarction; endocrine, for example, pregnancy, diabetic ketoacidosis; pancreatitis; end-organ failure, for example, renal **(uraemia), liver disease**, adrenal failure (Addison’s disease)
- Drugs: **ARVs** (e.g. **zidovudine, lopinavir/ritonavir**); antimicrobials (e.g. macrolides, beta-lactams, **TB drugs**); chemotherapy (oncology); digoxin; opioids; alcohol-intoxication, et cetera.

(Note: **Bold** indicates conditions that are frequent among the HIV-infected patients.)

**Management:** Address the cause. Inner-ear or motion sickness requires antihistamines, for example, dimenhydrinate, cyclizine; if drug-related – antidopaminergic, for example, prochlorperazine; if chemotherapy-related – 5-HT_3_ antagonist, for example, ondansetron, granisetron; if intestinal and gastro-related – 5-TH_4_ agonist, for example, cisapride, metoclopramide and somatostatin analogues, for example, octreotide. Chemotherapy-induced N&V may result in ‘anticipatory’ N&V viz. CNS-arousal and anxiety, and may require treatment with a benzodiazepine (e.g. lorazepam), glucocorticoids (e.g. methylprednisone), dexamethasone or with cannabinoids (e.g. tetrahydrocannabinol) (see Section 7).

Suggested end-of-life treatment:

- **Bowel obstruction**: Octreotide 100–200 µg subcutaneous injection 3 times a day or 100–600 µg per day in an IV infusion. Should a nasogastric tube (NGT) be placed? Where the likelihood is that this will provide little or no improvement and where the expectation is that death is within hours or a few days, it is more humane to withhold NGT and maximise alternative forms of symptomatic relief.
- Dexamethasone 4 mg po/IV–8 mg po/IV daily: maximum 16 mg daily.
- **Gastroparesis:**
- Metoclopramide 10 mg po/IV–20 mg po/IV every 4–6 h: maximum 100 mg per day.
- **Elevated intracranial pressure:**
- Dexamethasone 4 mg po/IV–8 mg po/IV daily: maximum 16 mg daily.
- **Unspecified cause but including chemotherapy**:
  ■ Metoclopramide 10 mg po/IV–20 mg po/IV every 4–6 h: maximum 100 mg per day.
  ■ Haloperidol Oral: 1.5 mg–5 mg 2–3X per day IV: 0.5 mg–2 mg every 8 h.
  ■ Ondansetron 8 mg po 8 h as needed.
  ■ Dexamethasone 4 mg po/IV–8 mg po/IV daily: maximum 16 mg daily. Usually combined with other anti-emetics.^52,53^

### Constipation

This is defined as infrequent emptying of bowel or rectum often with a sensation of incomplete evacuation. It is seldom secondary to a significant medical problem.

- **Acute or recent onset:** Local problem (viz. tumour, stricture, trauma or **analgesics including opioids, inactivity,** not eating).
- **Chronic:** Irritable bowel syndrome; hypothyroidism; hypercalcaemia; pregnancy; chronic CNS disorders (e.g. Parkinson’s disease, paraplegia et cetera); drugs (e.g. antidepressants, calcium channel blockers, **analgesics, including opioids**).

(Note: **Bold** indicates conditions that are frequent among the HIV-infected patients.)

**Management:** Treat the cause. Where feasible, encourage the patient to mobilise and take in more fluids and increase dietary fibre (bran) including vegetables and fruit. The following medicines can be tried: lactulose, psyllium, bisacodyl and fleet enemas. Management must be expectant and preventive.

Suggested end-of-life care:

- Senna 2–4 tabs (8.6 mg sennosides per tab) or 1–2 tabs as a single daily dose or in two divided doses per day. Do not exceed 100 mg per day.
- Bisacodyl suppository: 10 mg given rectally per day as needed.

### Fever

Fever in someone with HIV infection usually suggests an infectious complication. The constellation of fever, cough, weight loss and night sweats in an HIV-infected person in Africa indicates a heightened suspicion of tuberculosis. However, TB must be confirmed as it is a treatable, transmissible disease and its presence puts others at risk. Even at the end of life, TB must be diagnosed, treated and controlled and it is particularly important to check whether the disease is drug-resistant or not (Gene Xpert analysis gives information about rifampicin sensitivity or resistance). Even in the context of palliative care, patient isolation and infection control measures must be implemented.

If fever is part of an end-of-life infection, such as an aspiration pneumonia, the patient and his or her family may resist the idea of prolonging the inevitable with antibiotics and active interventions. The task of the palliative care team is to assist the patient to face the inevitability of death yet at the same time the team must agree to do all that it can to minimise suffering, that is, to support the patient. This is where the palliative care physician and team provide what is frequently missing from the wards of our South African hospitals as death should be about healing even when the latter is not delivered by way of pills, lines and procedures.

**Management:** Treat the cause when a fever is indicated. Paracetamol, aspirin, NSAIDS and sometimes steroids may assist in controlling it, while tepid sponging, a fan and oral fluids may help to alleviate the patient’s distress. Avoid paracetamol in patients with liver disease. Antimicrobial therapy that is not directed at a particular pathogen or a likely diagnosis is unlikely to be helpful at the end-of-life. Prophylactic trimethoprim sulfamethoxazole is recommended if the baseline CD4 count is < 350 c/mm^3^ and/or an AIDS-defining illness (or WHO stages 3 and 4 disease) is present.^50^

Suggested end-of-life care:

- Paracetamol/acetaminophen650 mg po/pr–100 mg po/pr or IV every 4–6 h as needed: maximum daily dose = 4 g.
- Naproxen 250 mg po bid–500 mg po bid (short course × 2–3 days. Can be repeated).

### Confusion and other neurological disease in people living with HIV.

Human immunodeficiency virus infects the human brain. This occurs during the first few weeks following acquisition of the virus and establishes a chronic but usually low-grade infection which persists for the remainder of the individual’s life. Neurological consequences are frequent but generally mild or asymptomatic and usually well controlled with ART. If the patient is naïve to ART or if the virus in the CNS has become resistant to ART, the function of the brain will be impaired for example, HIV encephalopathy. The latter is a life-threatening and dementing process characterised by motor-slowing, abnormal movement (basal ganglia involvement) and progressive cognitive decline. Human immunodeficiency virus also attacks the peripheral nervous system (PNS) and is responsible for a painful symmetric sensory polyneuropathy that leaves the individual wheelchair and bedbound and in constant pain. These conditions will usually respond to viral suppression with ARVs tailored to treat the CNS infection. Treatment with ARVs is warranted even in the context of palliation.

Several of the following conditions need to be considered in the HIV-infected patient with serious neurological disease,^54^ as detailed in Table 4.

**TABLE 4:** The association of certain cancers with HIV-1 infection in South Africa, 1995–2004.^63^

Cancer site or type	Total (*N*)	HIV-1 + (%)	Odds ratio (OR, 95% CI, adjusted for age, sex and year of diagnosis)
Kaposi’s sarcoma	333	89.2	50.4 (34.2–74.3)
Non-Hodgkin’s lymphoma	223	44.4	6.1 (4.4–8.4)
Squamous cell, skin	70	21.4	2.6 (1.4–4.7)
Anogenital other than cervix	157	22.3	2.5 (1.7–3.8)
Cervix	1586	14.9	1.7 (1.4–2.0)
Melanoma	53	15.1	1.6 (0.7–3.5)
Hodgkin’s lymphoma	154	19.5	1.5 (1.9–2.4)

*Source*: Stein L, Urban MI, O’Connell D, et al. The spectrum of human immunodeficiency virus-associated cancers in a South African black population: Results from a case-control study, 1995–2004. Int J Cancer. 2008;122:2260–2265. https://doi.org/10.1002/ijc.23391

Note: This table provides odds ratios that define the occurrence of various cancers in HIV-infected people in South African during 1995–2004. All of the cancers represented were associated with HIV infection rates greater than that of the general South African population of the time.

CI, confidence interval.

**Management:** Treat the cause where identified. Is the patient on ART (efavirenz)? TB drugs (Isoniazid)? Is the HIV infection controlled in the serum and in the CSF?

Suggested end-of-life care:

- Lorazepam for anxiety: 0.25 mg po or IV–2 mg po or IV or S/C every 4–6 h as needed. Increase the dose to 5 mg if necessary.
- Haloperidol for delirium: 0.5 mg po or IV–1 mg po or IV every hour as needed. When symptoms have settled, give the daily total dose in 3–4 divided doses through the day.

## HIV, cancer and palliative care in Southern Africa

Cancer case fatality rates are higher in low-income regions such as Africa (75%) than in high-income regions for example, Europe and North America (46%).^60^ On the back of this, the growing numbers of HIV-infected persons with cancer, a so-called ‘hidden cancer epidemic’ in LMICs, is of concern.^61,62^ More than a decade ago, South African researchers recorded the prevalence of several cancers among HIV-infected South Africans^63^ (see Table 5). Odds ratios confirmed a substantial increase in risk among the HIV-infected patients, particularly for the traditional AIDS-defining tumours, and in Africa these cancers still predominate: Kaposi’s sarcoma, non-Hodgkin’s lymphoma, cervical carcinoma and CNS lymphoma. The incidence of non-AIDS-defining cancers, however, in the region (e.g. Hodgkin’s disease, solid tumours of the lung, GIT and breast, et cetera) is rising despite the use of ART. The latter is a global trend and affects PLWHIV 10–15 years earlier than their peers with similar tumours.^64,65^

**TABLE 5:** Recommendations for cannabinoid use in symptom management of HIV/palliative care.^72^

Symptoms and recommended medication	Medication and/or evidence	Comment on medication and route of administration
**Nausea and vomiting**		
Dronabinol and the CBMs	Antiemetic effects when CB1 receptors activated by THC.	THC-rich products: Inhaled.
Dronabinol	-	Superior anti-emetic activity versus neuroleptics in cancer patients. Synergistic effect for dronabinol and prochlorperazine. Non-inferiority for dronabinol versus 5-HT3 antagonists.
CBMs’	-	Greater activity of CBMs in suppressing anticipatory nausea in pre-clinical model.
**Pain**		
Dronabinol	Dose: 10 mg better than 20 mg	THC-rich products
	Efficacy: Mild analgesic effect comparable to 60 mg codeine	THC/CBD 1:1
	Adverse reactions (20 mg): dizziness, somnolence, ataxia, blurred vision	Inhaled: Breakthrough pain/ pain crises = immediate benefit.
Nabiximols	Low dose (1–4 sprays/day) Medium dose (6–10 sprays/day) High dose (11–16 sprays/day)	Oral: Persistent pain (‘long-acting’ effect)
	-	Analgesia with low and medium dose versus placebo, poor drug tolerability with high dose
Natural cannabinoids	-	Reduction in pain intensity, opioid-sparing potential, synergism effect with opioids
	-	Improvement in pain measures with the use of cannabinoids compared with placebo. Benefit from the use of inhaled cannabis treatments for neuropathic pain. Prevention of chemotherapy-induced neuropathy in pre-clinical studies.
**Appetite stimulation**		
Dronabinol	Increased appetite and weight stability in HIV/AIDS and dementia	THC-rich products: Inhaled or oral
Natural cannabinoids versus megesterol acetate for cancer anorexia	-	Findings favour megesterol
THC efficacy per dose	THC 2.5 mg versus THC 2.5 mg + CBD 1 mg versus placebo	No significant improvements in survival, weight or other nutritional variables.
Smoked cannabis	-	Increased weight with smoked cannabis in experienced HIV+ marijuana smokers
Oral Dronabinol	-	Improved taste, smell and food enjoyment reported
**Insomnia**	
THC-rich products	Inhaled: Sleep induction Oral: Sleep maintenance	Association between cannabinoids and improved sleep quality
THC-rich products	Inhaled: Sleep induction Oral: Sleep maintenance	Lack of evidence in cancer and palliative care population
**Depression and anxiety**	
Nabiximols	High doses have negative effect in depression, positive results for anxiety disorders	Anxiety: CBD-rich Depression: THC-rich or THC/CBD 1:1 Inhaled: Panic attacks or anxiety
CBD-rich products	Recommended for patients with psychiatric disease	Oral: For persistent symptoms
THC	May exacerbate for example, schizophrenia, psychosis and bipolar disorder	-

*Source*: Cyr C, Arboleda MF, Kumar S, et al. Cannabis in palliative care: Current challenges and practical recommendations. Ann Palliat Med. 2018;7(4):463–477. https://doi.org/10.21037/apm.2018.06.04

THC, tetrahydrocannabinol; CBD, cannabinoid-drug.

Establishing early linkage to care, particularly to palliative and HIV care, is essential if the goal of improved survival and the relief of suffering is to be reached. However, several hurdles must be overcome:

- **Late presentation**. Stigma, misunderstanding and denialism continue as barriers to early detection of HIV and cancer. Patients present at an advanced HIV stage, naïve to ART, with a low baseline CD4 level and multiple competing diagnoses.
- **Delays in receiving cancer treatment.** Large number of patients, few or inadequately skilled staff, insufficient specialists and unsupportive management cause delay in treatment. In many instances, patients die before test results return and before a link with oncology and radiotherapy is made. Thirty African and Asian countries have no radiotherapy machines and in many LMICs, oncologists, if present, are restricted to the largest cities.^66^
- **Laboratory support.** Obtaining reports, for example, biopsy (histology) results, in the public sector in SA is plagued by long delays, while histology reports in the private sector return within a couple of days, and oncology referrals in the public sector cannot be processed without the confirmatory histology. In the absence of histological confirmation, patients die while awaiting their test results or while awaiting their oncology appointment.
- **HIV and cancer therapy.** Chemotherapy in Africa and SA’s public sector is frequently characterised by the ongoing use of drugs that are no longer viewed as optimal in developed regions.^67^ Problems include post-chemotherapy neutropenia, HIV immunosuppression, antimicrobial resistance, frequent drug stock-outs and drug–drug interactions between cytochrome P450 substrates, inducers and inhibitors, and ARVs.

How can the HIV-infected cancer patient be better served?

- **Prevention**
  ■ Tobacco use control
  ■ Hepatitis B vaccination
  ■ Hepatitis C serology and access to directly acting antivirals (DAAs)
  ■ Human papillomavirus (HPV) vaccination
  ■ Cervical and anal Pap smears
  ■ **The early introduction of ART.** Uncontrolled plasma (HIV) viral load is associated with the increased risk of malignancy in the HIV-infected cancer patients.^68,69^
- **Baseline determination of HIV status of every cancer patient.** Survival of the HIV and cancer patient requires access to ART and long-term suppression of HIV.^70,71^
- **Linkage to care.** The ethos of this care is holistic, that is, oncology, HIV-caregivers and the palliative care team work together in the support of the patient.
- **Collaboration is needed between oncology and radiotherapy and the disciplines of HIV/infectious diseases and palliative care.** Cancer care in SA must be sensitive to the needs of the HIV-infected people, and greater collaboration between HIV, palliative care physicians and oncology and radiotherapy specialists must take place if weak links in healthcare delivery are to be strengthened. Antiretroviral drugs and regimens are constantly changing, drug–drug interactions and toxicities are common, and secondary infection with opportunistic microbes frequently occurs. A team approach is required to improve the survival outcome in this group of patients.

## The cannabinoid drugs in the palliative management of HIV-infected patients.

South African courts have recently legalised cannabis for medical use. Data on HIV-infected people are sparse and restricted to observational reports, and although the use of these compounds is widespread both in Africa and globally, the hostility to its use is slowly changing. Nonetheless, data are beginning to emerge (see Table 5).^72^ An observational study in > 3000 cancer patients found that cannabis improved sleep, anxiety and depression levels and reduced the fatigue, nausea and vomiting caused by chemotherapy.^73^ A report of 198 HIV-infected ‘heavy’ cannabis users found reduced activation of inflammatory markers compared with non-cannabis using controls; however, there is clearly a need to better clarify the role of the cannabinoids in evidence-based, well-planned RCTs of the HIV-infected and uninfected people.^74^ The 2016 Johns Hopkins-Lancet Commission on Drug Policy and Health makes the additional point: ‘At a time of policy-level concern about dependence on prescription opioids, a few ecological studies suggest that greater access to cannabis could reduce use of opioids for pain relief’.^75^

Cannabinoid side effects are common, usually dose-dependent, that is, higher doses tend to produce more side effects, and are often specific to individual compounds (see Table 3). These side effects include cardiac (tachycardia, hypo- and hypertension), CNS (arousal and depression states, for example cognitive impairment, euphoria, psychosis and paranoia), and gastrointestinal toxicity such as diarrhea, vomiting and abnormal liver enzymes, with higher doses (see Table 6).^72^

With regard to the role of cannabinoids in the palliative care of HIV-infected patients, several ‘unknowns’ remain, for example^72^:

- Indications for use of cannabinoids require urgent clarification.
- The pharmacokinetics and pharmacodynamics of cannabinoids in people naïve to and those with prior exposure (to cannabinoids) in the context of palliative care. Does this differ? Do cannabinoid-exposed people require a higher dosing of cannabis?
- Drug–drug interactions between the cannabinoids, ART and TB drugs have minimal or no data.
- Which route of administration should be recommended: oral, inhaled (smoked)?

## Final remarks

It was the general belief in the 1980s that a vaccine and cure would have been found by the end of that decade or at the latest, that is, the middle of the 1990s. That did not occur, and the HIV epidemic is now firmly rooted in southern African soil. Antiretroviral therapy has transformed the infection into a chronic, manageable disorder yet the condition remains incurable. About 8 million HIV-infected South Africans need care and will die from or with the virus. Their suffering is the concern of these guidelines as many will require palliative care (see Box 3).

The ‘total pain’ that accompanies suffering arises from multiple causes. Analgesics alone do not effectively control this pain although palliative care teams throughout the country’s health service would go a long way to answer this need. Even highly motivated teams require funding, organisation and the support of colleagues and government in a country where its public health is in trouble: underfunded, overcrowded, ageing facilities in need of renewal and a department facing extraordinarily high levels of litigation.^77,78^

Developing a discipline of palliative care and fitting it into this failing system at this time will be a testing experience, but it must happen. Somehow.^75^

The private sector must find a reliable tool that funders can use when providing capital for home nursing and hospice admission. A life expectancy of 6 months or less is a widely used rule of thumb in the United States, although available models are generally insensitive to the gains of ART and the treatment of opportunistic disease. No local RCTs answer this question; nonetheless, our recommendation has been to follow the US approach and use the 6-month probability of survival or the SPICT^TM^ tool.

The defining treatment ethos of HIV and ID clinicians is curative. There is no conflict between curative and symptomatic management provided the goal to treat suffering is central to care. Is there a time where curative care is no longer appropriate? Is there a time to let go of ART? Yes. Those of us who are hospital-based clinicians encounter these questions daily in our wards and clinics. The answers are not usually found in textbooks but at the patient’s bedside.

## Appendix 1: Palliative care model: No care

Characteristics of this model include:

- **Acute hospitalisation**
  ■ Even when seriously ill, palliative care and support are generally not offered in public or private hospitals in South Africa.
  ■ Primary attention is directed to acute and curative care.
  ■ Care is seldom a team work; each caregiver works independently and very few have had formal training in palliative care.
  ■ The public health system’s masterplan is usually focused on early discharge and the emptying of beds in preparation for the next intake of acutely ill patients.
  ■ Patients are seldom, if ever, asked about their end-of-life thoughts or wishes.
  ■ Families rarely participate in the provision of the patient’s care.
  ■ The day-to-day care provided in many of SA’s public hospitals is given by junior doctors, for example, community service doctors, interns, medical officers, registrars, et cetera, particularly in district and rural hospitals. Supervision is not uniform and senior consultants in public hospitals are seldom in the wards every day and less often in the evenings and at night.
- **The HIV patient**
  ■ **Patient characteristics.** Many patients are newly diagnosed or previously diagnosed and started on ART but had been lost to follow-up. The presentation is diverse: comorbid conditions (e.g. diabetes, renal failure, hepatitis B, et cetera), AIDS-defining and non-AIDS- defining diseases (e.g. community acquired pneumonia [CAP], pneumocystis pneumonia [PJP], et cetera), anaemia, low platelets, gastroenteritis, ART-associated toxicity and treatment failure (viral resistance), et cetera. Occasionally the primary presentation is with a condition not immediately associated with HIV, for example, trauma, malaria, skin rash, et cetera. Often patients are very ill and the mortality rate is substantial.
  ■ **Tuberculosis** (TB) is frequent, needs to be excluded in all and is the major cause of death. Care requires attention to symptomatic control (palliation), curative therapy and the prevention of transmission.
  ■ **Poverty and limited social resources with adherence problems.** Non-disclosure to family members is frequent, stigma dictates behaviour and non-disclosure results in non-adherence which leads to treatment failure. Neurocognitive dysfunction is common and may contribute to non-adherence (forgetfulness) and unreliable clinic attendance of some. The care of the HIV-infected needs to be broad-based to ensure that social, psychological and practical needs are met.
  ■ **The HIV-sick.** Many patients present with more than one major diagnosis, for example, TB and bacterial pneumonia, PJP and renal failure, Kaposis’s sarcoma (KS) and bacteraemia et cetera, and multiple concurrent diagnoses increase mortality. Antiretrovirals are toxic and drug–drug interactions are frequent and occasionally life-threatening, for example, drug-induced liver injury (DILI), Stevens–Johnson syndrome (SJS) and erythema multiforme (EM).
- **Laboratory indices**
  ■ **Baseline HIV diagnostic tests** (HIV antibody tests) are still not performed on all who are admitted to South African private and public hospitals. HIV-directed care, for example, ARVs, cannot be given without this baseline information. Only ART can control HIV infection.
  ■ **CD4 counts** are often low in this group of patients, for example, < 200 cells/mm^3^, and patients are vulnerable to a variety of opportunistic diseases.
  ■ **Viral load.** The South African National ART guidelines discourage checking a patient’s **viral load (VL)** at the time of the baseline assessment, but recommend that the VL is checked 6 months after starting ART and annually thereafter. Elevated VL means adverse survival if on ART. Persistently high VL indicates increased risk of cancer in the HIV-infected people.
  ■ **Markers of mortality:** Anaemia (Hb < 8 g/dL), CD4 < 200 cells/mm^3^, VL (especially > 100 000 copies/mL), end-organ failure (renal, liver), a low BMI (< 18 kg/m^2^).
  ■ **Delay in laboratory turn-around times.** Histology results appear particularly resistant to short turn-around times yet the care of **the HIV-cancer patient** depends on tissue confirmation. Patients without timely results do not get chemotherapy, do not start ART early enough and do not survive.
- **Radiology**
  ■ Access to ultrasound, echocardiography, computed tomography (CT) and magnetic resonance imaging (MRI) scans, et cetera, in the government sector and especially in rural and secondary level hospitals is poor and delays in obtaining these tests are widespread, even in larger public hospitals.
  ■ Transfer to referral centres is difficult to expedite: transport is not always readily available and permission is needed, as is the willingness of the referral hospital to accept the patient.
- **Medical team**
  ■ The care of the HIV-well is explained in guidelines. The care of the HIV-sick requires practical training and the acquisition of bedside skill, as good palliative care will require a good knowledge of the care of the HIV-sick.
  ■ Junior doctors, for example, community service doctors, interns and registrars, carry a major responsibility in the day-to-day provision of patient care in SA’s public hospitals. Additional training will be needed to equip junior staff on how to triage between the curative and palliative disciplines of care and the combinations of both.
- **Dying in a South African public hospital**
  ■ Impending death is often accompanied by feelings of terror, loneliness, pain and abandonment. These feelings are likely to add to the suffering of patients in busy hospitals characterised by shortages of beds, staff and medicines. Family often absent at the time of death. The ‘end-of-life’ discussion occurs rarely, if at all, and the family often feels left-out and aggrieved, and regret occurs as seldom the gathering together of loose ends takes place during the contented arrival of life’s end.
- **Bereavement counselling**
  ■ Usually not given to the patient or family before or after death. Questions of the bereaved are often left unaddressed and anger and disappointment are seldom addressed. Litigation is an option for the disgruntled family.

## Appendix 2: Indicators of increased mortality of HIV-infected patients

**Demographic indicators of an increased risk of death:**

- Age ≥ 45–50 years old. Risk increases with age and includes risk from comorbid disease. Male gender is frequently associated with increased risk but confounded by late entry into care, poor retention in care, lower baseline CD4 cell counts and older age at entry than female gender.

**Clinical indicators of an increased risk of death:**

- Unintended weight loss of > 10% of normal body weight, and/or BMI ≤ 18 kg/m^2^.
- AIDS-defining clinical conditions yet the risk is not the same for all events. Tuberculosis, cryptococcal meningitis (CM) and malignancy in Africa carry a higher risk than other AIDS-defining conditions.
- AIDS and non-AIDS-defining cancers in the HIV-infected people.
- Comorbid disease and end-organ failure: renal, liver, cardiac and respiratory.
- Confirmed ARV-resistance, particularly high-level resistance to all major classes of ARV.
- Hospital re-admission rates and Grip Strength. Thirty-day hospital re-admission rates in HIV-positive adults are associated with other markers of poor outcome, namely low CD4 counts, AIDS-defining illnesses, non-AIDS-defining infections and unreliable utilisation of medication.

**Laboratory indicators of an increased risk of death:**

- CD4 count: CD4 ≤ 200c/mm^3^ or lower. The lower the level (e.g. < 50 c/mm^2^), the greater the risk.
- Anaemia: particularly if severe (viz. Hb < 8 g/dL).
- HIV viral load: high levels (viz. > 100 000 copies/mL) prior to ART and detectable levels while on ART. The higher the level, the greater the risk, for example, malignancy.

**Combination indicators of an increased risk of death:**

- The Veterans Aging Cohort Study (VACS) Index. This scoring system developed in the USA incorporates seven clinical or laboratory indices that, taken together, predict death in HIV-infected persons. Several observational reports indicate that this index has superior predictive value to both single and composite indices currently in use. The role of Hepatitis C virus positivity in the VACS scoring system is numerically small and unlikely to negatively influence its utility in Southern Africa where the prevalence of HCV is low, that is, 3% – 5%.

## Appendix 3: The Karnofsky score

The Karnofsky score^28^ was developed in 1948, enabling physicians to evaluate a patient’s ability to survive cancer chemotherapy. It is still in general use; however, its insensitivity and non-specificity with regard to HIV-infected patients limit its use in deciding on access to hospice-related care.

- 100 – Normal; no complaints; no evidence of disease
- 90 – Able to carry on normal activity; minor signs or symptoms of disease
- 80 – Normal activity with effort; some signs or symptoms of disease
- 70 – Cares for self; unable to carry out normal activity or to do active work
- 60 – Requires occasional assistance, but is able to care for most of their personal needs
- 50 – Requires considerable assistance and frequent medical care
- 40 – Disabled; requires special care and assistance
- 30 – Severely disabled; hospital admission is indicated although death not imminent
- 20 – Very sick; hospital admission is necessary; active supportive treatment is necessary
- 10 – Moribund; fatal processes progressing rapidly
- 0 – Dead.

*Source:* Karnofsky DA, Abelmann WH, Craver LF, Burchenal JH. The use of the nitrogen mustards in the palliative treatment of carcinoma – With particular reference to bronchogenic carcinoma. Cancer. 1948;1(4):634–656. https://doi.org/10.1002/1097-0142

## Appendix 4: The Support and Palliative Care Indicator Tools (SPICT^TM^)

The SPICT^TM^ is a guide for identifying people at risk of deteriorating health and imminent death.^29^ This group of patients must be assessed for unmet supportive and palliative care needs.

**1. Look for two or more general indicators of deteriorating health:**

- Performance status is poor or worsening, that is, the person is in bed or in a chair for ≥ 50% of the day; reversibility is judged as limited
- Dependent on others for most care needs; dependency is because of physical and/or mental health problems
- Two or more unplanned hospital admissions in the past 6 months
- Significant weight loss (5% – 10%) over the past 3–6 months and/or a low BMI < 18 kg/m^2^
- Persistent, troublesome symptoms despite optimal treatment of underlying conditions
- Patient asks for supportive and palliative care or for the withdrawal of treatment.

**1. Look for any clinical indicators of one or more advanced conditions:**

**Cancer:**

- Functional ability deteriorating because of progressive metastatic cancer
- Too frail for oncology treatment or treatment is for symptom control only.

**Dementia/frailty:**

- Unable to dress, walk or eat without help
- Eating and drinking less; swallowing difficulties
- Urinary and faecal incontinence
- No longer able to communicate using verbal language; minimal social interaction
- Fractured femur; multiple falls
- Recurrent febrile episodes or infections; aspiration pneumonia.

**Neurological disease:**

- Progressive deterioration in physical and/or cognitive function despite optimal therapy
- Speech problems with increasing difficulty communicating and/or progressive swallowing difficulties
- Recurrent aspiration pneumonia; breathless or respiratory failure.

**Heart and/or vascular disease:**

- New York Heart Association (NYHA) Class III/IV heart failure or extensive, untreatable coronary artery disease with:
  ■ Breathlessness or chest pain at rest or on minimal exertion
- Severe, inoperable peripheral vascular disease.

**Respiratory disease:**

- Severe chronic lung disease with:
- Breathlessness at rest or on minimal exertion between exacerbations
- Needs long-term oxygen therapy
- Has needed ventilation for respiratory failure or in whom ventilation is contraindicated.

**Kidney disease:**

- Stage 4 or 5 chronic kidney disease (viz. an eGFR < 30 mL/min), with deteriorating health
- Kidney failure complicating other life-limiting conditions or treatments
- Discontinuation of dialysis.

**Liver disease:**

- Advanced cirrhosis with one or more complications in the past year:
  ■ Diuretic-resistant ascites
  ■ Hepatic encephalopathy
  ■ Hepatorenal syndrome
  ■ Bacterial peritonitis
  ■ Recurrent variceal bleeds
- Liver transplantation is contraindicated.

**What to do: Review supportive and palliative care planning:**

- Review current treatment and medication so that the patient receives optimal care.
- Consider referral for specialist assessment if symptoms or needs are complex and difficult to manage.
- Agree to current and future care goals and a care plan with the patient and his or her family.
- Plan ahead if the patient is at risk of loss of capacity.
- Record, communicate and coordinate the care plan.

*Source:* The Support and Palliative Care Indicator Tools (SPICT^TM^) [homepage on the Internet]. University of Edinburgh, SPICTTM; 2015.[cited 03 Nov 2019] Available from: http://www.spict.org.uk

## Appendix 5: The established Veterans Aging Cohort Study index scoring scheme

The VACS Index utilises age, routine laboratory markers such as CD4 count, HIV-1 RNA, haemoglobin, platelets, AST and ALT, creatinine and markers of liver impairment (viz. FIB-4 and HCV status).

**TABLE 1-A5:** A Restricted VACS index. The risk of mortality of PLWHIV on at least 12 months of ART increases with a rising VACS score. (Max. vacs score= 164).^30^

Component	Level	VACS index points assigned
Age (years)	< 50	0
	50–64	12
	≥ 65	27
CD4 count (cell/mm^3^)	≥ 500	0
	350–499	6
	200–349	6
	100–199	10
	50–99	28
	< 50	29
Viral load (copies/mL)	< 500	0
	500-log1 × 10^5^	7
	≥ log1 × 10^5^	14
Haemoglobin (g/dL)	≥ 14	0
	12–13.9	10
	10–11.9	22
	< 10	38
FIB-4†	< 1.45	0
	1.45–3.25	6
	> 3.25	25
eGFR (mL/min) ‡	≥ 60	0
	45–59.9	6
	30–44.9	8
	< 30	26
Hepatitis C co-infection	Yes	5
	No	0

*Source*: Tate JP, Justice AC, Hughes MD, et al. The VACS index: An internationally generalizable risk index for mortality after one year of antiretroviral therapy. AIDS. 2013;27(4):563–572. https://doi.org/10.1097/QAD.0b013e32835b8c7f

VACS, Veterans Aging Cohort Study.

† , FIB-4 = age (year) × AST/ platelet in 100/L × √ALT.

‡ , eGFR = 186.3 × (creatinine)^−1.154^ × (age) ^−0.203^ × (0.742 for women) × (1.21 for black individuals).

## Appendix 6: Palliative care management: Drugs used for symptom control in HIV palliation

**TABLE 1-A6:** Palliative care management, drug–drug interactions.

Symptom	Palliative drug treatment	Antiretroviral interaction	Potential hazards
**Fatigue and weakness**	-	-	Where possible treat the underlying cause and not just the symptom
	Corticosteroids	Entry inhibitor(EI): None Nucleoside/tide inhibitor (NRTI):	None
		None	None
		Non-nucleoside inhibitor (NNRTI) (CYP450 enzyme induction): Minor interaction	None
		Integrase inhibitor (INSTI): None	None
		Boosted protease inhibitor (bPI): Potential interaction	Long-term co-administration with bPIs = potential toxicity of steroid (viz. Cushing’s syndrome and/or worsening of HIV-related immune suppression)
	Methylphenidate	None	None
	Pemoline	None	None
	Dextroamphetamine	EI: None	None
		NRTI: None TDF=tenofovir difumarate, FTC=emtricitabine	None
		NNRTI: None Integrase inhibitor	None
		(INSTI):DTG = dolutegravir, RTG/RAL = raltegravir, ETG = elvitegravir, cob = cobisitat	None with RAL and DTG, but avoid with ETG/ cob +TDF+FTC
		bPI: None	None
	Modafinil	EI: Interaction likely with MVC	None
		NRTI: None	None
		NNRTI: Potential interaction RPV = rilpivirine NVP = nevirapine	Modafinil = weak enzyme inducer. Do not use with RPV, caution with NVP
		INSTI: None	None
		bPI: Potential interaction	Potential for increased toxicity of modafinil with the bPIs: use with caution
**Weight loss and anorexia**	-	-	Where possible treat the underlying cause and not just the symptom
	Corticosteroids	As above	As above
	Androgenic steroids	As for steroids above EI: None	None
		NRTI: None	None
		NNRTI: None	None
		INSTI: None	None
		bPI: Small potential for toxicity	Long term use = potential for androgenic toxicity with long term use of bPI
	Oxandrolone	None	None
	Megestrol acetate	None	None
	Dronabinol	EI: None NRTIs: None NNRTIs: Efavirenz (EFV) and Etravirine (ETR) = Caution INSTI = None bPIs = potential decrease in activity of dronabinol	None None EFV and ETR inhibit CYP2C9 and to a lesser extent 3A4 and may increase dronabinol toxicity; Ritonavir (low-dose) is a mild enzyme inducer = reduce dronabinol activity
	Growth hormone	None	None
**Fever/Sweats**	-	-	Where possible treat the underlying cause and not just the symptom
	NSAIDS: Anti-inflammatory effect	EI: None NRTIs: None NNRTIs: Start with lowest dose of aspirin, ibuprofen, et cetera. NNSTIs: None bPIs: None	None Renal toxicity and risk of bleeding (platelet dysfunction). Caution with TDF + renal disease. EFV and ETR inhibit CYP2C9 = potential for bleeding. Use with caution None None
	Corticosteroids	As above	As above
	Anticholinergics: diphenhydramine, biperiden, chlorpromazine et cetera.	EI: None NRTIs: None NNRTIs: None INSTIs: None bPIs: Caution	None None None None bPIs block CYP2D6 and may inhibit the metabolism of many anticholinergic drugs; Monitor the patient carefully
	H2 Antagonists: cimetidine ranitidine	EI: None NRTIs: None NNRTI: Caution with RPV bPI: Caution with ATV Must give max dose 400 mg ATV+ 100 mg ritonavir if using TDF backbone!	NB: Reduced absorption of H2 antagonists, for example, ranitidine, in the absence of gastric acid; give RPV 4 h before ranitidine or 12 h after; in the presence of TDF, always give boosted ATV/r as absorption is otherwise compromised
**Gastrointestinal**			
Nausea and Vomiting (N&V)	-	-	Where possible treat the underlying cause and not just the symptom
	Drug-related. Dopamine antagonists: Haloperidol	EI: None NRTIs: None NNRTIs: Caution INSTIs: None bPIs: Caution	Haloperidol levels decreased with NVP, EFV and ETR; potential for prolongation of QT interval with RPV, haloperidol and the PIs = risk of Torsáde de Pontes
	Prochlorperazine	EI: None NRTIs: Caution. Risk of marrow suppression with AZT (ZDV) and prochlorperazine NNRTIs: Caution bPI: Caution	Caution: Risk of QT prolongation with prochlorperazine, RIL and bPIs; ECG monitoring recommended
	Drug-related. Opiates: Metoclopramide	None	None
N&V associated with abdominal distention	Antihistamines: Promethazine	EI: None NRTI: None NNRTI: None INSTI: None bPI: Caution, potential toxicity	Promethazine metabolised via CYP2D6; if inhibited, may potentiate toxicity.
N&V associated with GIT obstruction	Anticholinergics: Scopolamine and hyoscine	None	None
N&V associated with raised intracranial pressure	Corticosteroids	Above	Above
N&V associated with chemotherapy, radiotherapy and/or surgery	Serotonin-antagonists: Granisetron, ondansetron, dolasetron	EI: None NRTI: None NNRTI: Caution with all, but watch for QT interval prolongation with RIL: Caution INSTI: none bPI: May potentiate toxicity. Caution.	None None All metabolised via CYP3A4; All NNRTIs may decrease levels e.g. RPV. Granisetron/ ondansetron prolong QT interval; bPIs potentiate toxicity of these agents
N&V associated with pre-vomit anxiety	Benzodiazepines: lorazepam	EI: None NRTI: None NNRTI: None INSTI: None bPI: Avoid with midazolam, oxazepam and lorazepam – Safe	Co-administration of bPI with midazolam may potentiate toxicity and result in fatal respiratory suppression
**Diarrhea**	-	-	Where possible treat the underlying cause rather than just the symptom
	Bismuth	None	None
	Methylcellulose	None	None
	Kaolin	None	None
	Diphenoxylate + atropine	None	None
	Octreotide	EI: None NRTI: None NNRTI: Caution INSTI: None bPI: Caution	Octreotide utilises CYP3A4: NNRTI is expected to decrease octreotide levels; bPI use is expected to enhance toxicity of octreotide
	Opiates	Above	Above
**Constipation**	Lactulose	None	None
	Senna	None	None
	Bisacodyl	None	None
**Respiratory**			
Dyspnoea, Shortness of Breath	-	-	Where possible treat the underlying cause and not just the symptom
	Opiates	Above	Above
	Bronchodilators	None	None
	Methylxanthines	None	None
	Benzodiazepines	Above	Above
**Cough**	Suppressants Opiates: Codeine, dextromethorphan	Above	Above
	Decongestants	None	None
	Expectorants	None	None
**Pre-agonal airways buildup of secretions: Gurgling, ‘Death-rattle’**	Atropine	None	None
	Hyoscine	None	None
	Scopolamine: Transdermal	None	None
	Glyco-pyrrolate	None	None

*Source*: Merlin et al.^33^; World Health Organization^37^; Blinderman and Billings^38^; Portenoy^40^

**TABLE 2-A6:** Palliative care seizure management, drug–drug interactions.

Drug	Advantage	Disadvantage	Interaction
Carbamazepine	Effective, low cost	Enzyme inducer	Reduces levels of NNRTI and PI: Avoid
Phenobarbital	Effective, low cost	Enzyme inducer	Reduces levels of NNRTIs and PI: Avoid
Phenytoin	Effective, low cost	Enzyme inducer	Reduces levels of NNRTIs and PI: Avoid
Sodium Valproate	Effective, useful for migraine, mood stabiliser	Enzyme inhibitor	May increase AZT toxicity: Caution; can be used with other ARVs
Gabapentin	Effective for neuropathic pain	Useful for control of focal seizures	No drug–drug interactions
Lamotrigine	Effective against focal and generalised seizures, help with bipolar depression	Effective against all types of seizures	Susceptible to enzyme inducers, for example, rifampicin: Caution
Levetiracetam	Effective, well-tolerated, more expensive	Useful for all types of seizure	No drug interactions

*Source*: Merlin et al.^33^; World Health Organization^37^; Blinderman and Billings^38^; Portenoy^40^

**TABLE 3-A6:** Palliative care pain management, drug–drug interactions.

Medication	Comment
**Non-opioid Analgesics**	
Paracetamol	Exercise caution when using the NNRTIs, NVP and EFV. and the boosted PIs with high doses or prolonged courses of paracetamol particularly in patients with known hepatic risk factors e.g. active HBV/HCV infection, concurrent anti-tuberculosis medication, alcohol abuse et cetera. Drug-induced liver injury (DILI) is a frequent diagnosis in HIV wards.
NSAID: Ibuprofen	No specific ARV interaction but check baseline renal, hepatic and bleeding risk in view of the potential for platelet dysfunction
Opioid analgesics	No specific interaction is anticipated with EIs, NRTIs and INSTIs. The risk of decreased opiate activity with NNRTI interaction is minimal but must be considered if pain control is difficult to achieve. Caution is advised when prescribing opioids with boosted protease inhibitors e.g. lopinavir/ritonavir, as a small risk of potentiating opiate toxicity exists.
**Tricyclic antidepressants**	
Amitriptyline, Desipramine	A small risk of prolongation of the QT interval exists with concurrent use of rilpivirine (RPV). A baseline ECG is recommended in those with risk factors for cardiac disease and those on anti-arrhythmic drugs and the anti-tuberculosis drugs, bedaqualine and linezolid.
Selective Serotonin/noradrenalin reuptake (SNRIs) Duloxetine/Venlafaxine	There is a small risk of potentiating the toxicity of these agents with bPIs. Caution and starting with lowest possible effective dose levels is recommended.
NMDA receptor antagonists Ketamine	Concurrent use of the NNRTIs and/or the bPIs may reduce or increase ketamine levels respectively. Caution is indicated.
Pregabalin and Gabapentin	There are no anticipated drug-drug interactions with the currently available ARVs.

*Source*: Merlin et al.^33^; World Health Organization^37^; Blinderman and Billings^38^; Portenoy^40^.
